# Understanding a Single-Li-Ion COF Conductor for Being Dendrite Free in a Li-Organic Battery

**DOI:** 10.34133/2022/9798582

**Published:** 2022-10-02

**Authors:** Yongjiang Sun, Genfu Zhao, Yao Fu, Yongxin Yang, Conghui Zhang, Qi An, Hong Guo

**Affiliations:** School of Materials and Energy, Yunnan University, No. 2, Green Lake North Road, Kunming 650091, China

## Abstract

In addition to improving ion conductivity and the transference number, single-Li-ion conductors (SLCs) also enable the elimination of interfacial side reactions and concentration difference polarization. Therefore, the SLCs can achieve high performance in solid-state batteries with Li metal as anode and organic molecule as cathode. Covalent organic frameworks (COFs) are leading candidates for constructing SLCs because of the excellent 1D channels and accurate chemical-modification skeleton. Herein, various contents of lithium-sulfonated covalently anchored COFs (denoted as LiO_3_S-COF1 and LiO_3_S-COF2) are controllably synthesized as SLCs. Due to the directional ion channels, high Li contents, and single-ion frameworks, LiO_3_S-COF2 shows exceptional Li-ion conductivity of 5.47 × 10^−5^ S · cm^−1^, high transference number of 0.93, and low activation energy of 0.15 eV at room temperature. Such preeminent Li-ion-transported properties of LiO_3_S-COF2 permit stable Li^+^ plating/stripping in a symmetric lithium metal battery, effectively impeding the Li dendrite growth in a liquid cell. Moreover, the designed quasi-solid-state cell (organic anthraquinone (AQ) as cathode, Li metal as anode, and LiO_3_S-COF2 as electrolyte) shows high-capacity retention and rate behavior. Consequently, LiO_3_S-COF2 implies a potential value restraining the dissolution of small organic molecules and Li dendrite growth.

## 1. Introduction

Due to the high mechanical strength and inflammable features of solid-state electrolytes (SSEs), the solid-state Li metal batteries matched cathode materials with highly specific capacity and SSEs can achieve high-energy density and safety properties. Therefore, SSEs play a crucial role in realizing solid-state batteries [1–5]. Among various SSEs, single-Li^+^ conductors (SLCs) show high ion conductivity and transference number. Moreover, the polarization of concentration difference can be addressed because there are no randomly removable anion and solvent in SLCs. Notably, the multiple species of freely mobile anion and solvent molecule inevitably trigger ununiform Li^+^ deposition and dendrite formation on the Li metal surface [6–9]. Consequently, designing and constructing advanced SLCs without anion and solvent are urgently needed.

Two-dimensional covalent organic frameworks (COFs) are popular crystalline materials and show a larger surface area, adjustable skeleton, and easily chemical modification [10–15]. Significantly, the active groups or units are effectively locked into the skeleton of COFs, satisfying a variety of applications [16–21]. These outstanding characteristics provide an ideal setting for creating Li^+^ conductors with high ion conductivity and transference number [22–24]. Consequently, COFs have been intensively investigated as advanced Li^+^ SSEs in recent years [25–34].

Although COF-based SLCs have been reported and studied such as TpPa-SO_3_Li with ion conductivity of 2.7 × 10^−5^ S · cm^−1^ and transference number of 0.9 [32] as well as LiCON-3 with ion conductivity of 3.21 × 10^−5^ S · cm^−1^ and transference number of 0.92 [33], the ion conductivity and transference number are still far below expectations. The main reason is that finite active site is anchored onto COFs, generating a lower Li^+^ content [35, 36]. Besides, these Li^+^-conducting COFs are established by injecting Li salts or solvent molecules into the frameworks (Figure S1a and 1b), resulting in anion-caused interfacial side reactions, which impedes the Li^+^ movement [37–39]. Furthermore, the multicomponent of solvent and anion in the COF-based SSEs might generate ambiguously interfacial reaction mechanism [40–42]. Therefore, it is critically necessary to accurately modify and design a multiactive center for increasing ion conductivity and transference number.

**Figure 1:**
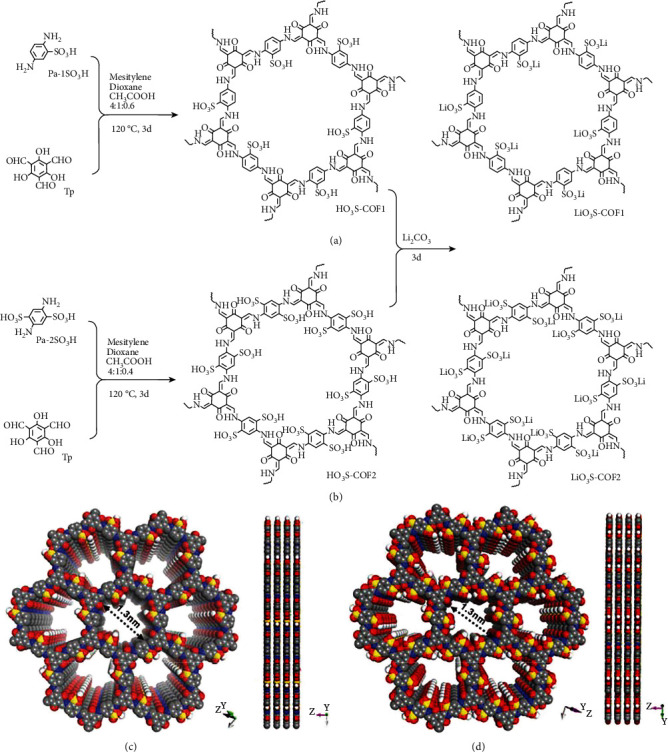
The synthetic route for LiO_3_S-COF1 (a) and LiO_3_S-COF2 (b); structural representations of the AA stacking mode of LiO_3_S-COF1 (c) and LiO_3_S-COF2 (d) (O: red; N: blue; C: grey; S: yellow; Li: white).

Herein, we designed various COF-based SLCs by covalent modification of active-site SO_3_Li into the pores of COFs without additional padding Li salts (Figure S1c). The synthetic route and chemical structure of single-ion COF conductors (LiO_3_S-COF1 and LiO_3_S-COF2) are provided in Figure 1. Particularly, the anionic framework could avoid the interfacial side-reaction and restrain dendrite growth. As a result, LiO_3_S-COF2 permits stable Li^+^ plating/stripping in Li/Li symmetric battery. Furthermore, the Li^+^ migration's dynamics and distance are improved. It leads to big ion conductivity and small activation energy. The Li^+^ migration behaviors are fully deduced by DFT theoretical calculation. Moreover, the electrolyte is successfully implemented in solid-state organic Li battery. This strategy can hinder Li dendrite growth and avoid the organic cathode dissolution, leading to excellently cyclic and rate performances. We therefore anticipate that this study will lead to the development of a new technology that will enable COF-based single-ion conductors in solid-state batteries.

## 2. Results and Discussions

### 2.1. Structural and Morphological Characterizations

As shown in Figures 1(a) and 1(b), LiO_3_S-COF1 and LiO_3_S-COF2 are synthesized by condensation 1,3,5-triformylphloroglucinol (Tp), 2,5-diaminobenzenesulfonic acid (Pa-1SO_3_H),, and 2,5-diaminobenzene-1,4-disulfonic acid (Pa-2SO_3_H) at mixed solvents of mesitylene, 1,4-dioxane, and CH_3_COOH. Subsequently, the sulfonic acid groups are easily reacted with Li^+^ to form lithium-sulfonated COFs of LiO_3_S-COF1 and LiO_3_S-COF2. Both the intermediate products of HO_3_S-COF1 and HO_3_S-COF2 are connected by the C-N bond. Therefore, the characteristic peak of C-N is detected by the Fourier transform infrared (FT-IR) spectrum. Figure S2–S3 display obvious peaks for HO_3_S-COF1 and HO_3_S-COF2. It is ascribed to the stretching vibration of C-N [43]. The C-N bonds indicate successful condensation reaction between −CHO and −NH_2_ groups and complete conversion into COFs. Moreover, the complete chemical conversion is further proved by the C=C bonds (1572 cm^−1^ for HO_3_S-COF1 and 1576 cm^−1^ for HO_3_S-COF2) [44, 45].

**Figure 2:**
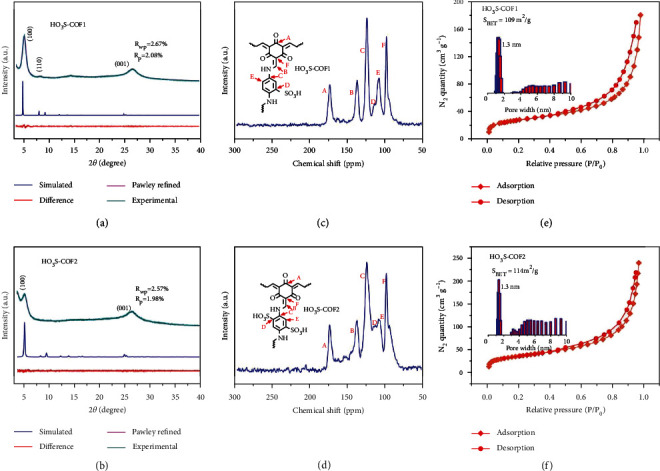
PXRD patterns with experimental, simulated Pawley refined results of HO_3_S-COF1 (a) and HO_3_S-COF2 (b); solid-state ^13^C NMR spectra of HO_3_S-COF1 (c) and HO_3_S-COF2 (d); N_2_ sorption isotherm and pore size distribution of HO_3_S-COF1 (e) and HO_3_S-COF2 (f).

The crystallinity of obtained HO_3_S-COF1 and HO_3_S-COF2 is investigated with powder X-ray diffraction (PXRD) measurements. For HO_3_S-COF1, Figure 2(a) shows three obvious peaks at 2*θ* of 4.76, 8.05, and 26.4°. They are attributed to the (100), (110), and (001) faces [43]. For further study structure, Pawley refinement is implemented. Low values of *R*_wp_ = 2.67% and *R*_p_ = 2.08% (Figure 2(a), pink) suggest high in accordance with experiment (Figure 2(a), green). In comparison with alternative AA and AB models, the HO_3_S-COF1 adopts the AA stacking (Figure S4). No peaks of starting materials of Tp and Pa-1SO_3_H appear in HO_3_S-COF1 (Figure S5), demonstrating complete conversion. Consequently, the synthesis of the targeted sample of LiO_3_S-COF1 is implemented by suspending the HO_3_S-COF1 and Li_2_CO_3_. The LiO_3_S-COF1 (Figure S6) has similar PXRD with HO_3_S-COF1 after replacement of H atom by Li^+^. For the HO_3_S-COF2, two obvious peaks at 4.49 and 26.12° can be observed in Figure 2(b). The two peaks are attributed to (100) and (001) faces [46, 47]. Moreover, the Pawley refinement (Figure 2(b), pink) with low values of *R*_wp_ = 2.57% and *R*_p_ = 1.98% indicates a good structure consistent with experimental data (Figure 2(b), green). HO_3_S-COF2 has AA stacking structure (Figure S7). No peaks of starting materials of Tp and Pa-2SO_3_H exist in the HO_3_S-COF2 (Figure S8). And the PXRD pattern of lithium-sulfonated LiO_3_S-COF2 has a similar profile to HO_3_S-COF2 (Figure S9). Moreover, the solid-state ^13^C NMR for HO_3_S-COF1 (Figure 2(c)) and HO_3_S-COF2 (Figure 2(d)) can further prove the C=O at ~174 ppm and C-N bond at ~137 ppm. The solid-state ^13^C NMR result is consistent with the aforementioned analyses, indicating the successful preparation of sulfonated COF materials.

The porosity of prepared COFs is investigated by N_2_ sorption/desorption isotherms. The samples of HO_3_S-COF1 and HO_3_S-COF2 show a surface area of 109 and 114 m^2^·g^−1^ according to N_2_ sorption/desorption isotherms at 77 K (Figures 2(e) and 2(f)), respectively. The HO_3_S-COF1 and HO_3_S-COF2 (Figures 2(e) and 2(f) inset) exhibit a micropore feature with a cavity size of 1.3 nm. The value is in high accordance with the simulated model (Figures 1(c) and 1(d)). X-ray photoelectron spectroscopy (XPS) is applied to detect the element species of COFs. The XPS spectra of S 2p of COFs (Figure S10–13) suggest the complete structure of COFs. Moreover, the Li 1s for LiO_3_S-COF1 and LiO_3_S-COF2 (Figure S14–15) reveals that the LiO_3_S-COF2 sample has higher Li content than that of the LiO_3_S-COF1. The Li content can be further proved by inductively coupled plasma detection for LiO_3_S-COF1 (2.01 wt%) and LiO_3_S-COF2 (2.89 wt%). Thermogravimetric analysis (TGA) of LiO_3_S-COF1 and LiO_3_S-COF2 (Figure S16) indicates high thermal stability.

**Figure 3:**
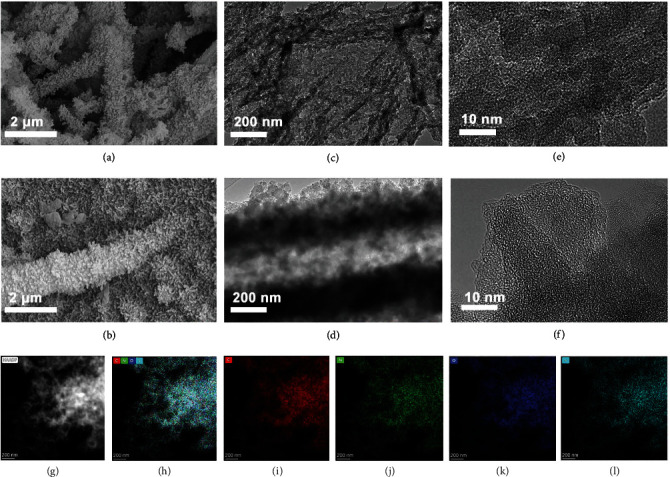
SEM images of HO_3_S-COF2 (a) and LiO_3_S-COF3 (b); TEM images of HO_3_S-COF2 (c) and LiO_3_S-COF2 (d); HRTEM images of HO_3_S-COF2 (e) and LiO_3_S-COF2 (f); EDS mappings of LiO_3_S-COF2 (g–l).

The microstructures of prepared COFs are studied by a scanning electron microscope (SEM) and transmission electron micrographs (TEM). Wirelike micrographs of HO_3_S-COF1 can be found in SEM and TEM images (Figure S17a–d). After lithiation, the wirelike morphology is not changed (Figure S17b–d) and the high-resolution TEM of the LiO_3_S-COF1 image shows a smooth structure, indicating no Li metal nanoparticles or clusters on the LiO_3_S-COF1 surface. The EDS mappings of LiO_3_S-COF1 can also suggest that the elements are uniformly distributed (Figure S17g–l). For the HO_3_S-COF2 and LiO_3_S-COF2, a similar morphology can be observed in SEM and TEM images (Figures 3(a)–3(f)). In addition, the C, N, O, and S elements are uniformly distributed (Figures 3(g)–3(l)). No obvious micrograph changes of the prepared COF before and after lithiation suggest outstanding structure stability.

### 2.2. Electrochemical Performances

**Figure 4:**
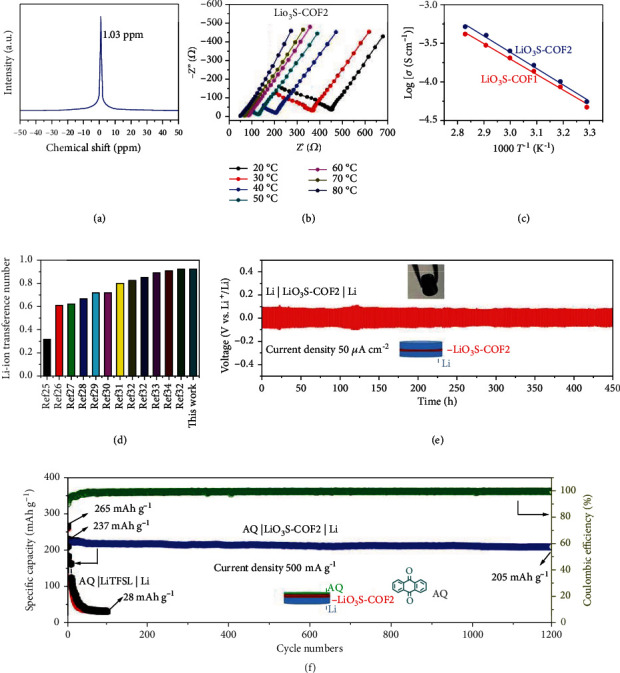
Solid-state ^7^Li NMR characterization of LiO_3_S-COF2 solid-state electrolyte (a); EIS measurements made over a range of temperatures from 20 to 80°C (b); the Arrhenius plot of ionic conductivity as a function of temperature for LiO_3_S-COF1 and LiO_3_S-COF2 (c); comparison of the Li-ion transference number of our study with other works (d); Li stripping-plating test of Li | LiO_3_S-COF2 | Li at the current density of 50 *μ*A·cm^−2^ for 1 h per cycle (e); cycling performance of AQ | LiO_3_S-COF2 | Li and AQ | LiTFSI | Li batteries at the current density of 500 mA·g^−1^ (f).

According to the above result, the SLCs of LiO_3_S-COFn are successfully synthesized. In order to detect the chemical environment of Li^+^ in LiO_3_S-COF2, the solid-state ^7^Li NMR spectrum is used and illustrates a singlet at 1.03 ppm (Figure 4(a)). This result proves an equivalent chemical environment [33, 34]. In addition, the single peak demonstrates Li^+^ dissociation, fast diffusion, and migration [48]. The electrochemical performance for LiO_3_S-COF2 is studied. The self-standing pellet is obtained by the cold-pressing method. Electrochemical impedance spectroscopy (EIS) is employed to investigate the Li^+^ transportation ability of LiO_3_S-COF2. Nyquist plots of LiO_3_S-COF2 are recorded under various temperatures (20 to 80°C) (Figure 4(b)). The EIS curve shows a semicircular profile made by plots of real component (Z) versus the imaginary component (Z^″^). Based on curves, the calculated resistances of LiO_3_S-COF2 are 450, 365, 202, 81, 62, and 40 *Ω* at 20, 30, 40, 50, 60, 70, and 80°C, respectively. Accordingly, the investigated Li^+^ conductivities are 4.46 × 10^−5^, 5.47 × 10^−5^, 9.9 × 10^−5^, 1.63 × 10^−4^, 2.47 × 10^−4^, 3.23 × 10^−4^, and 5.06 × 10^−4^ S · cm^−1^ at 20, 30, 40, 50, 60, 70, and 80°C, respectively, according to the impedance result and equation (described in the Supporting Information). It is worth noticing that the LiO_3_S-COF2 shows a high Li^+^ conductivity of 5.06 × 10^−4^ S · cm^−1^ at 80°C.

For the LiO_3_S-COF1, the EIS is displayed in Figure S18. It is obviously found that LiO_3_S-COF1 exhibits larger resistances than LiO_3_S-COF2. Consequently, the calculated Li^+^ conductivity at 20°C is 2.67 × 10^−5^ S · cm^−1^ according to impedance results as shown in Table S1. According to the Arrhenius plots (Figure 4(c)), LiO_3_S-COF2 and LiO_3_S-COF1 show low Ea value of 0.15 eV and 0.18 eV, respectively. The obtained small Ea is adjacent to other Li^+^ conductors, certifying the directional Li-ion migration channel. The Li^+^ transference number (*t*_Li_^+^) is measured to be 0.93 for LiO_3_S-COF2 and 0.91 for LiO_3_S-COF1 (Figure S19) at 20°C using potentiostatic polarization means, demonstrating Li^+^ contribution to the ion conductivity [49]. Besides, the electrochemical window is studied by the linear sweep voltammetry (LSV). The LSV is recorded under a sweep rate of 10 mV · s^–1^ in a voltage range from 0.5 to 6.0 V (vs. Li/Li^+^) at room temperature. As demonstrated in Figure S20, the current density keeps constant until the voltage is a higher than 4.3 V for LiO_3_S-COF2. Thus, LiO_3_S-COF2 exhibits an electrochemical window of 4.3 V than LiO_3_S-COF1 (4 V) within published work [33], indicating a wide range of working voltage. The Ea, Li^+^ conductivity, and *t*_Li_^+^ for LiO_3_S-COF1 and LiO_3_S-COF2 are compared and displayed in Table S1. The LiO_3_S-COF2 conductor shows better electrochemical behaviors than LiO_3_S-COF1, mainly caused by the more active-center content on the LiO_3_S-COF2. More notably, the comparison of Li^+^ conductivity, Ea, and *t*_Li_^+^ indicates distinctly better electrochemical performance for LiO_3_S-COF2 than other reported materials (Table S2). Compared with Li-ion conductors with propylene carbonate (PC), ethylene carbonate (EC) solvent, and Li salt (Table S2), LiO_3_S-COF2 presents remarkable Li^+^ conductivity. This is because LiO_3_S-COF2 has numerous easily accessible sites and well-designed directional Li-ion channel. LiO_3_S-COF2 has Higher *t*_Li_^+^ than others (Figure 4(d)), implying that it has outstanding single-Li^+^-conducting behavior.

We further assess the application of LiO_3_S-COF2 in solid-state Li metal batteries. The prepared LiO_3_S-COF2 SSE film shows high flexibility (exhibited in Figure 4(e) inset). As shown in Figure 4(e), the Li metal electrode assembled by symmetric lithium metal battery configuration (inset of Figure 4(e)) is made to assess the practical use of the LiO_3_S-COF2 conductor. So, the galvanostatic Li plating/stripping is studied under a current density of 50 *μ*A·cm^−2^ for 1 h each cycle. Figure 4(e) demonstrates stable Li plating/stripping behaviors more than 450 h without obvious fluctuation of potential. The prominent result is caused by the single-Li-ion-conducting behavior. The stability of the framework for LiO_3_S-COF2 after electrochemical measurement is investigated by PXRD and FT-IR. From the FT-IR (Figure S21) and PXRD (Figure S22) patterns before and after the test, no obvious changes can be observed. This result implies that LiO_3_S-COF2 has excellent skeleton stability.

The potential application of LiO_3_S-COF2 is further evaluated by constructing an organic battery. Universally, the organic carbonyl cathodes cause plentiful attentions in green electrode for rechargeable Li-ion batteries [50–52], as these compounds show eco-friendly and renewable great preponderance. However, the dissolution of this electrode material in liquid electrolyte will inevitably cause fast decay of specific capacity and hinder cyclability and rate behaviors. This issue could be resolved by fabricating solid-state batteries. Therefore, the performance of LiO_3_S-COF2 is further evaluated in the Li-organic battery. The organic battery is made of Li metal as anode and anthraquinone (AQ) as cathode. The AQ has ultrahigh capacity in traditional LIBs. Nevertheless, the capacity of AQ cathode decays distinctly and shows poor performances. This issue can be worked out in the solid-state LIBs [33]. Therefore, we assemble AQ | LiO_3_S-COF2 | Li solid cell and measure its electrochemical performances. Liquid electrolyte (10 *μ*L LiPF_6_ in EC/DEC *v*/*v* = 1 : 1) is added to the electrode surface to improve interface contact [30]. Figure S23 exhibits the galvanostatic charging-discharging curves at a current density of 500 mA·g^−1^. The cathode AQ displays a discharging-specific capacity of 228 mAh·g^−1^, lightly lower than the liquid cell AQ | LiTFSI | Li with 278 mAh·g^−1^. Additionally, the AQ | LiO_3_S-COF2 | Li has better rate behaviors (Figure S24) and cycling stability (Figure 4(f)) than AQ |LiTFSI| Li battery (Figure 4(f) and Figure S25). The excellent cycling stability of AQ|LiO_3_S-COF2|Li is caused by restraining the dissolution of AQ cathode in liquid electrolyte.

**Figure 5:**
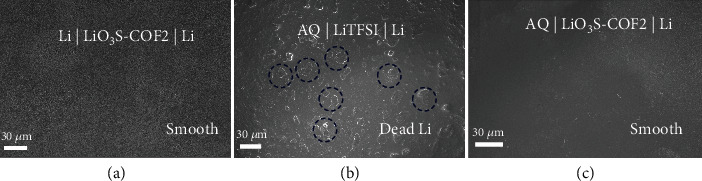
SEM images of the surface for Li metal in Li | LiO_3_S-COF2 | Li (a), AQ | LiTFSI | Li (b), and AQ | LiO_3_S-COF2 | Li batteries (c).

Li dendrite growth and dead Li on the Li metal electrode surface are the major threats and challenges of Li metal batteries. Therefore, the surface morphology of Li metal electrodes in a symmetric battery, quasi-state-solid battery, and liquid battery is investigated by SEM technique. As shown in Figure 5(a), for the Li metal surface of symmetric battery Li| LiO_3_S-COF2 |Li, no dead Li can be observed. The result suggests that Li-ion is uniformly deposited to the surface of metal Li in LiO_3_S-COF2. For the AQ |*LiTFSI*| Li liquid battery, the inhomogeneous Li deposition is obviously observed by SEM after cycling (Figure 5(b)). That is because there is no functional material to guide the uniform Li-ion flux (Figure S26a). However, due to the well-defined channels, COF materials can achieve uniform Li^+^ deposition (Figure S26b). Hence, no dead Li or dendrite after cycles for AQ|*LiO*3*S* − *COF*2|Li is supported by SEM (Figure 5(c)), implying homogeneous Li^+^ deposition. The aforementioned phenomenon demonstrates that LiO_3_S-COF2 displays well-done single-Li-ion conductor behavior, which can avoid unwanted side reactions [37–40]. As a consequence, the single-Li-ion conductor LiO_3_S-COF2 has potential value in a solid-state Li metal battery.

### 2.3. Mechanism Studies

**Figure 6:**
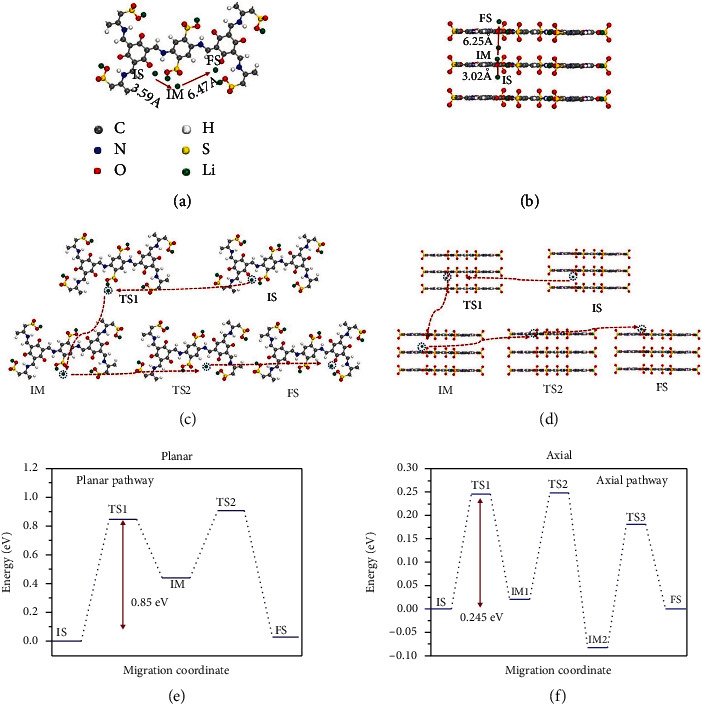
Theoretical illustration of Li^+^ migration behaviors inside the planar (a) and axial pathways (b); detailed theoretical elucidation of Li^+^ migration behaviors inside the planar and axial pathways (c, d); migration barriers for planar (e) and axial pathways (f), respectively.

The density functional theory (DFT) calculation is carried out to study the Li-ion migration routes in LiO_3_S-COF2. LiO_3_S-COF2 is a 2D-extended material. Generally, two routes are parallel and perpendicular for Li^+^ migration, which are divided into planar and axial approaches (Figures 6(a) and 6(b)), respectively. Different pathways for Li^+^ migration are expounded by evaluation of migratory barriers at rate-determining steps for planar and axial. Figure S27 shows the optimized Li^+^ geometries. The oxygen atom of keto-form groups in COF promotes for the migration of Li^+^ via the cation-dipole interaction [53, 54]. As shown in Figures 6(d) and 6(f), axial shows a lower migratory barrier of 0.245 eV than planar of 0.85 eV (Figures 6(c) and 6(e)). Some conditions are beneficial for Li^+^ migration with axial. Firstly, the pore of LiO_3_S-COF2 results in an entered condition than interplanar distance. The pore size (1.3 nm) of LiO_3_S-COF2 is larger than the Li^+^ radius (0.076 nm) than interplanar distance (0.48 nm), as shown in Figure S27. Therefore, Li-ion migrates in the pore. Secondly, the distance of the axial pathway is shorter than the planar pathway (Figure S28). Beyond all question, the C=O groups provide electrostatic interaction and accelerate the dissociation of −SO_3_Li, which plays an important role for promoting Li^+^ migration. To sum up, the above theoretical analyses and experimental results demonstrate that Li^+^ directionally transports across the stacked channel of LiO_3_S-COF2 under the reliable assistance of O atoms and the *π*-electronic system.

## 3. Conclusion

In conclusion, a novel lithium-sulfonated covalent organic framework is successfully prepared as single-Li^+^ SSE. The active center of −SO_3_Li is covalently tethered into the 1D porous channel of COF to achieve single-ion behaviors. The unique framework structure and abundant active centers are beneficial for Li^+^ migration. Thus, LiO_3_S-COF2 has high conductivity and transference number. Li-ion migration behaviors and routes are thoroughly studied and illustrated using DFT. Based on directional ion channel, the Li-ion conductor has exceptional electrochemical performance in quasi-solid-state batteries. This strategy can solve the crucial issue of the organic cathode dissolution in liquid electrolyte. Therefore, our work might accelerate the advancement of COF-based solid-state electrolyte.

## 4. Materials and Methods

### 4.1. Chemicals

The chemical reagents and characterized apparatus are described in the supporting information.

### 4.2. Electrochemical Measurements

All the electrochemical measurements are provided in the supporting information.

## Data Availability

The data used to support the findings of this study are included within the article and the supplementary information file.

## Supplementary Materials

Supplementary Materials The chemical reagents, characterized instruments, electrochemical tests, DFT calculation details, tables, and other materials are provided in the supporting information.
