# Supplementary material for: Understanding a Single-Li-Ion COF Conductor for Being Dendrite Free in a Li-Organic Battery
Source: Research (Wash D C). 2022 Oct 2;2022:9798582. doi: 10.34133/2022/9798582 (PMC9575471; doi:10.34133/2022/9798582)
Supplement: Supplementary Materials — The chemical reagents, characterized instruments, electrochemical tests, DFT calculation details, tables, and other materials are provided in the supporting information. [file 9798582.f1.zip › Supporting Information.docx]

**Supporting Information**

**Understanding Single Li-Ion COF Conductor for Dendrite-Free in Li-Organic Battery**

Yongjiang Sun, Genfu Zhao*, Yao Fu, Yongxin Yang, Conghui Zhang, Qi An, Hong Guo*

School of Materials and Energy, Yunnan University, No. 2, Green Lake North Road, Kunming 650091, China

Correspondence should be addressed to Genfu Zhao; fu1165158114@126.com and Hong Guo; guohong@ynu.edu.cn

**S1 Materials**

All the starting materials and solvents, unless otherwise noted, are used without purification. 1,3,5-Triformylphloroglucinol (Tp), 2,5-diaminobenzenesulfonic acid (Pa-1SO_3_H) and 2,5-diaminobenzene-1,4-disulfonic acid (Pa-2SO_3_H) were purchased from the Jilin Chinese Academy of Sciences-Yanshen Technology Co., Ltd. Dioxane, mesitylene, CH_3_COOH (6.0 M), Li_2_CO_3_ and anthraquinone (AQ) were obtained from Shanghai Titan Scientific Co. Ltd. HO_3_S-COF1 was synthesized according to previous literature.^1^

**S2 Characterized instruments**

The crystallinity of obtained COFs was measured by powder X-ray diffraction (PXRD) and recorded using a Bruker D8 Advance X-ray diffractometer at 40 kV and 40 mA over 2° to 40° 2*θ* range with a Cu Kα radiation. The microstructures of prepared materials were studied by scanning electron microscope (SEM) (AMRAY 1000B) and transmission electron micrographs images (TEM, JEM-2010). The content of Li in LiO_3_S-COFn (n=1,2) was measured by inductively coupled plasma (ICP) analysis using Inductively Coupled Plasma Atomic Emission Spectrometer (Prodigy, The U.S.A). The porosity of HO_3_S-COF1 and HO_3_S-COF2 were investigated by nitrogen adsorption–desorption isotherms measured at 77 K on an Autosorb IQ2 absorptiometer (Quantachrome Instruments). The thermostability of LiO_3_S-COF1 and LiO_3_S-COF2 were measured by thermogravimetric analysis (TGA) carried out on a Netzch STA449F3 analyzer under N_2_ atmosphere at a heating rate of 10 °C/min from 25 to 800 °C. X-ray photoelectron spectroscopy (XPS) test was conducted to demonstrate the chemical component in an Escalab 250Xi instrument from Thermo Scientific equipped with an Al Kα microfocused X-ray source, and the C1s peak at 284.8 eV was used as internal standard. Solid-state nuclear magnetic resonance (NMR) experiments were conducted using an Agilent VNMRS 600 MHz NMR spectrometer at room temperature. All the processes of electrochemical measurements were implemented by using a Princeton 2273 electrochemical workstation. The galvanostatic discharge/charge tests were carried out by using Neware CT-4008-5V50mA-164 laboratory instrument in the voltage range of 1.5-4.0 V (vs. Li^+^/Li) at different current densities.

**S3 Electrochemical measurements**

Ionic conductive: Ionic conductivities were measured with an Li-ion blocking titanium (Ti)/LiO_3_S-COFn/Ti (n=1,2) symmetric cell based on an electrochemical impedance spectroscopy (EIS) analysis at a frequency range from 10^–2^ to 10^6^ Hz and an applied amplitude of 10 mV using a Bio-logic VSP classic potentiostat. The ionic conductivities (σ) are determined according to following equation:

σ=L/ZA

where L is the thickness of pellet sample, Z is the electrode area (cm^2^) and the A is the impedance (Ω). The activation energy (Ea) is determined from the slope of the Arrhenius plot.

Li-ion transference number: Li-ion transference number (t_Li_⁺) was evaluated using a potentiostatic polarization method at room temperature.^2^ The DC current flowing through the Li/LiO_3_S-COF2/Li symmetric cell and the AC impedance of the cell before and after polarization were measured to determine the t_Li_⁺ value of LiO_3_S-COFn according to following equation:

*t*_Li⁺_ $=\frac{Iss(\triangle V-I0R0)}{I0(\triangle V-IssRss)}$

where I0 is the initial current, I00 represents the steady-state current, ∆V symbolizes the applied potential, R0 and RSS are the interfacial resistances before and after the polarization, respectively.

Linear sweep voltammetry (LSV) was conducted with a Ti/LiO_3_S-COFn/Li asymmetric cell operated under a sweep rate of 10 mV s^–1^ in a voltage range from 0.5 to 6.0 V (vs. Li/Li^+^) at room temperature.

**S4 Preparation of LiO_3_S-COFn solid electrolyte film**

Solid electrolyte films of LiO_3_S-COFn for EIS using were obtained by adding ~15 mg of the LiO_3_S-COFn powder to a stainless-steel die (10.0 mm) and then pressed at ca. 250 MPa for 40 min in an Ar-filled glovebox (Mikrouna) with O_2_ < 0.1 ppm and H_2_O < 0.1 ppm. The thickness ca. 160 μm of film was measured by a calliper. Solid electrolyte films of LiO_3_S-COF2 for battery using were obtained by adding ~15 mg of the LiO_3_S-COF2 powder to a stainless-steel die (13.0 mm) and then pressed at ca. 250 MPa for 40 min in an Ar-filled glovebox (Mikrouna) with O_2_ < 0.1 ppm and H_2_O < 0.1 ppm. The thickness ca. 120 μm of film was measured by a calliper.

**S5 Preparation of AQ cathode material**

For the AQ cathode, the AQ powders, Ketjenblack and PVDF were mixed at a weight ratio of 60:30:10 in NMP solvent to form a homogeneous slurry and then coated on Al foil. The electrodes were dried at 80 °C for 12 h under vacuum. It was then punched into discs (Φ = 12 mm). The active cathode materials loading was about ~0.9 mg cm^–2^. The quasi-solid-state cells were assembled with the following structure: AQ | LiO_3_S-COF2 film | Li. Liquid electrolyte (10 μL LiPF_6_ in EC/DEC v/v=1:1) was added to the electrode surface (Lithium metal anodes and AQ cathodes) to improve interface contact.^3^ For the comparison, the liquid cell was constructed as following structure: AQ | liquid electrolyte | Li. The galvanostatic charge/discharge tests were performed in a battery testing system (using Neware CT-4008-5V50mA-164 laboratory instrument) with the cut-off voltages of 1.5–4.0 V.

**S6 Density Functional Theory (DFT) Calculations**

The unit cell structure of LiO_3_S-COF2 has been established, and it has been optimized based on density functional theory (DFT) using a 1 × 1 × 2 supercell model with 202 atoms. First-principles calculations was performed using density functional theory (DFT)^4^ within project augmented wave method (PAW)^5^ implemented in the Vienna ab initio Simulation Package (VASP).^6^ Exchange and correlation energy is performed by Perdue-Burke-Ernzerhof (PBE)^7^ version of the generalized gradient approximation (GGA). The hydrogen atoms of carboxylic acids were substituted into Li atoms and geometry optimization was performed to determine their stable locations using a 3 × 1 × 1 supercell model with 357 atoms. The Brillouin zone is sampled using 1 × 1 × 1 based on Monkhorst-Pack k-point mesh. A plane-wave cutoff energy of 450 eV is efficient to guarantee the convergence of the total energy. The atoms are completely relaxed with force below than 0.01 eV/Å. Furthermore, to determine the diffusion energy barrier and the minimum energy pathways for Li diffusion, the climbing-image nudged elastic band (CI-NEB) method was performed.^8^ First of all, the most stable initial state and final state of Li for axial and planar models are optimized between two layers of COF. It was noting that the carboxylic group had some rotation to stabilize the Li atom, coordinated with three O atoms. And Li atom has two O ligands in the intermediate state. Four points are inserted between the two states for axial model. Eight and two points are inserted between initial and IM, IM and final state respectively.

**S7. Synthesis of** **HO_3_S-COF1***.*

At a Pyrex tube were added by Tp (63.0 mg, 0.3 mmol), Pa-1SO3H (68.5 mg, 0.45 mmol). Then, a mixed 3 mL solution of mesitylene/1,4-dioxane (4:1, v/v) was added to the tube. Subsequently, the tube was sonicated for 15.0 min. Then, an aqueous solution of 0.6 mL CH_3_COOH (6.0 M) was added to the mixture. The tube was frozen in a liquid N_2_ bath and sealed under a vacuum. After sealing, the tube was placed in an oven at 120 ºC for 3 d. After cooling at room temperature, the yielded solid was collected by filtering and washed with tetrahydrofuran (THF) and acetone several times, respectively. Solid was further purified by acetone for 1 d. Finally, after dried at 120 °C for 12 h under vacuum, HO_3_S-COF1 was obtained as red powder.

**S8. Synthesis of HO_3_S-COF2.**

At a Pyrex tube were charged by Tp (63.0 mg, 0.3 mmol), Pa-2SO3H (120.0 mg, 0.46 mmol). Then, a mixed 3 mL solution of mesitylene/1,4-dioxane (4:1, v/v) was added to the tube. Subsequently, the tube was sonicated for 15.0 min, and CH_3_COOH (0.4 mL, 6.0 M) was added to the mixture. The tube was frozen in a liquid N_2_ bath and sealed under a vacuum. After sealing, the tube was placed in an oven at 120 ºC for 3 d. After cooling at room temperature, the yielded solid was collected by filtering and washed with tetrahydrofuran (THF) and acetone for several times, respectively. Solid was further purified by acetone for 1 d. Finally, after dried at 120 °C for 12 h under vacuum, HO_3_S-COF2 was obtained as red powder.

**S9. Synthesis of** **LiO_3_S-COFn***.* In typical process, the above synthesized COFs (HO_3_S-COF1, HO_3_S-COF2) (100.0 mg) were suspended in an aqueous solution of Li_2_CO_3_ (20.0 mL, 0.5 M) and stirred for 3 d at the round-bottom flask, respectively. Subsequently, the resultant solid was collected by filtration and washed with deionized water several times in order to remove excess Li_2_CO_3_. Finally, the solid was dried at 100 °C for 1 d under vacuum to yield pure functional LiO_3_S-COFn.


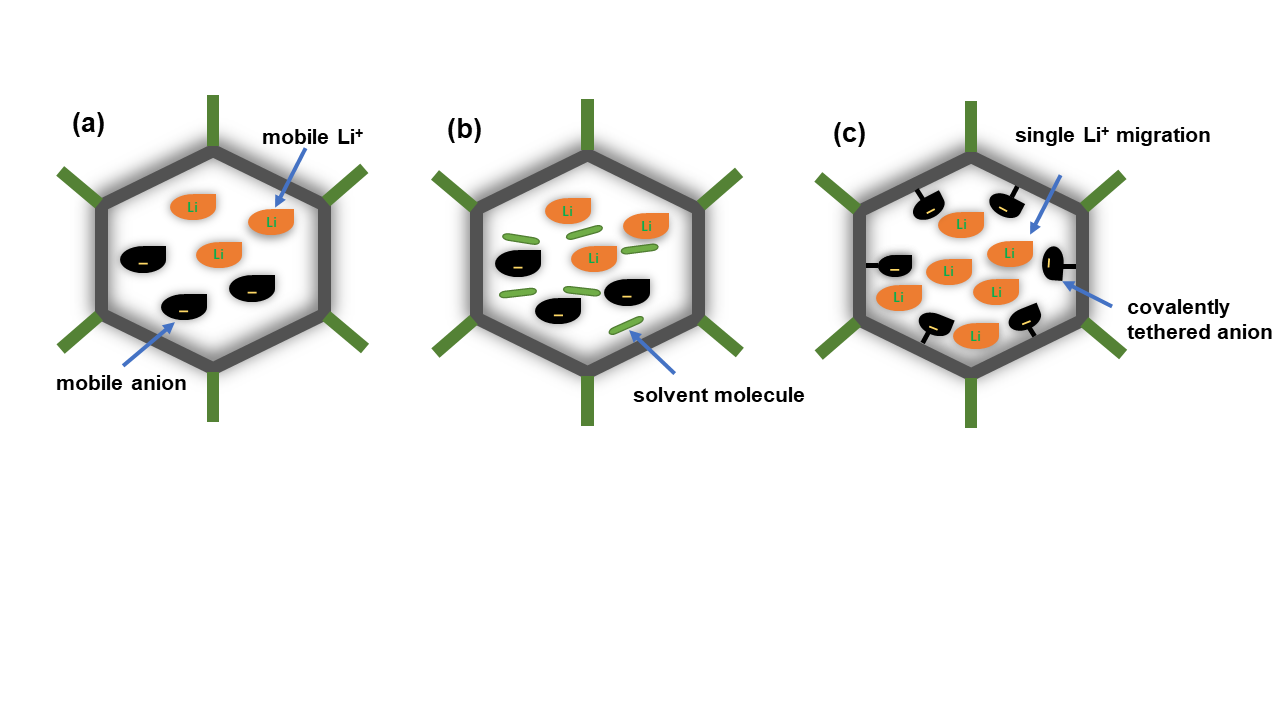


**Figure S1**. Graphical representation of Li^+^ conducting COFs materials with previous work based on addition of Li salts or organic solvent (a and b); our study based on solvent-free and single Li^+^ conducting COF.


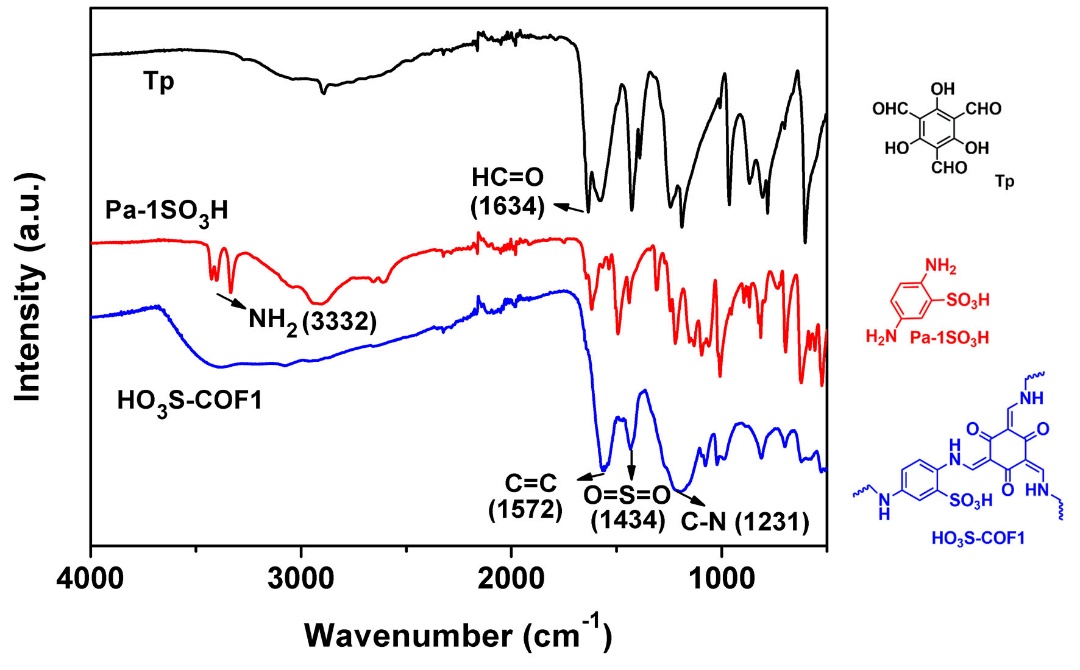


**Figure S2**. FT-IR spectra of starting materials of Tp, Pa-1SO_3_H and as-prepared HO_3_S-COF1, respectively.


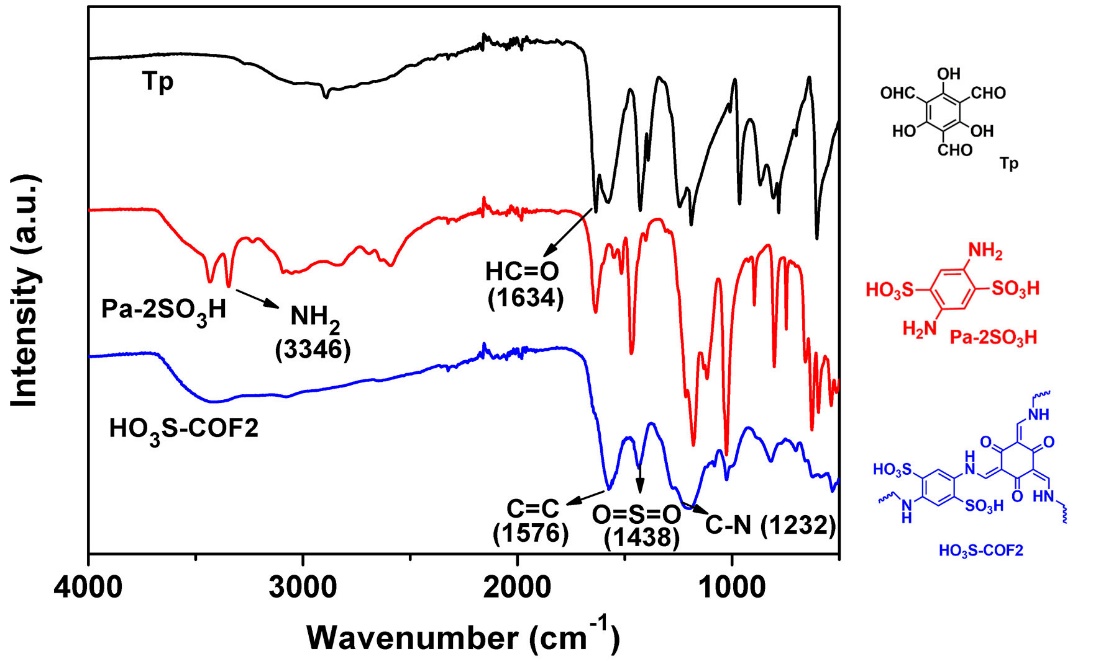


**Figure S3**. FT-IR spectra of starting materials of Tp, Pa-2SO_3_H and as-prepared HO_3_S-COF2, respectively.


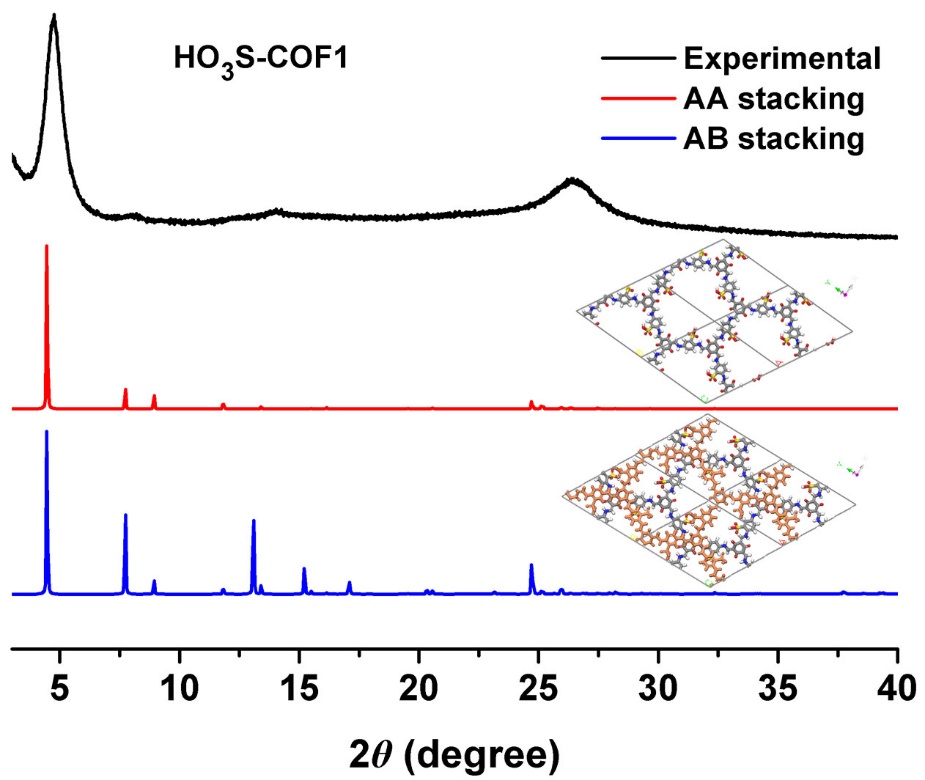


**Figure S4**. Calculated PXRD patterns based on the AA stacking, AB stacking and experimental result for HO_3_S-COF1.


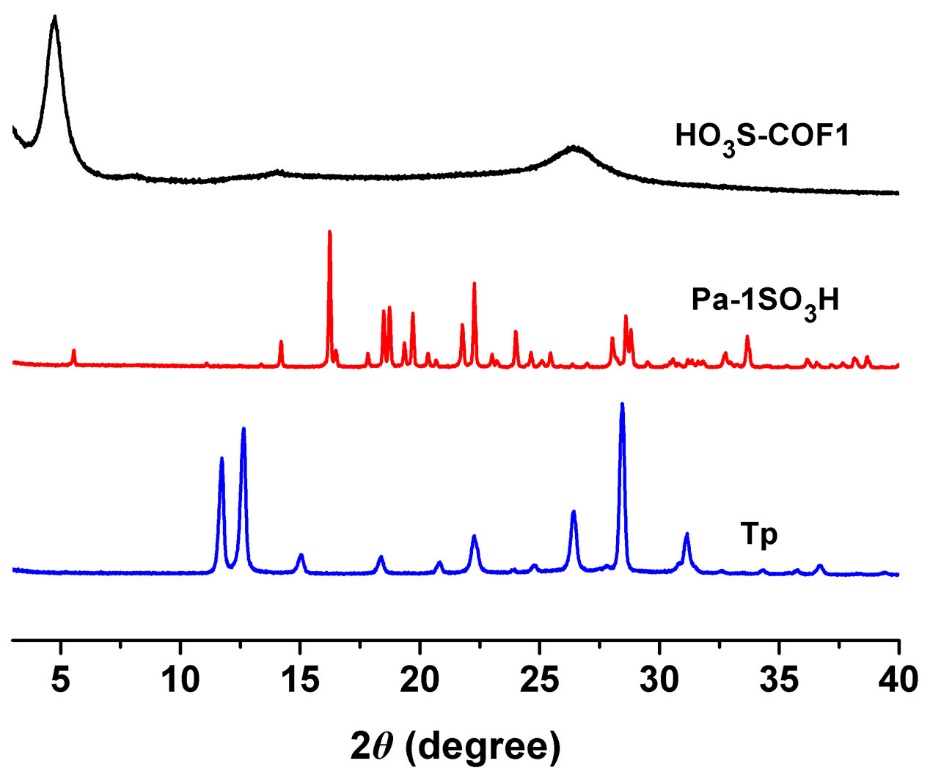


**Figure S5**. PXRD patterns of HO_3_S-COF1 and starting materials of Tp and Pa-1SO_3_H.


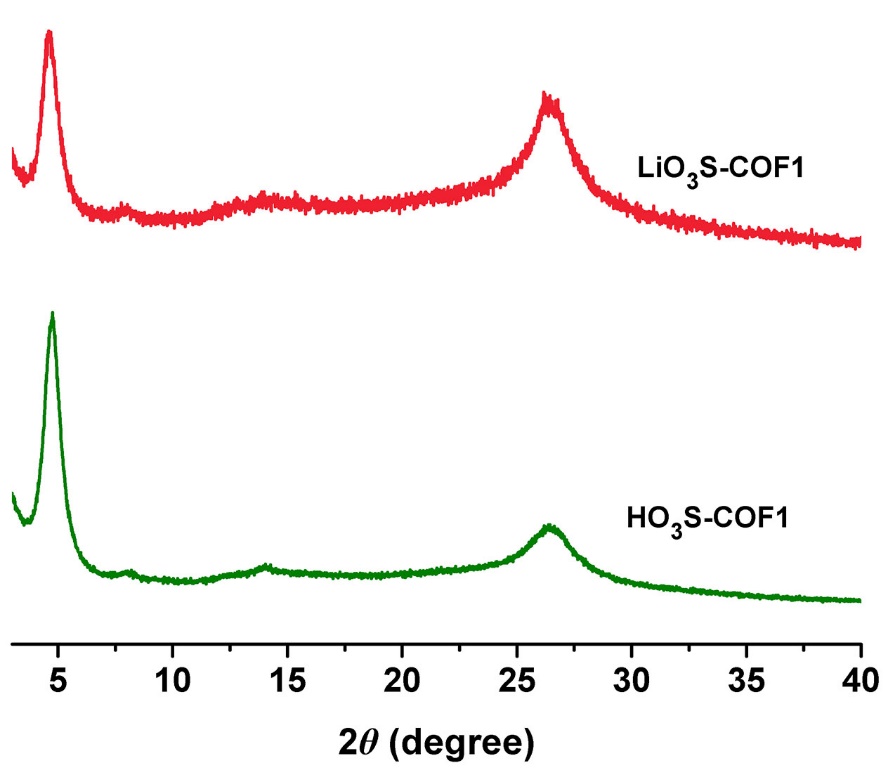


**Figure S6**. PXRD patterns of HO_3_S-COF1 and LiO_3_S-COF1.


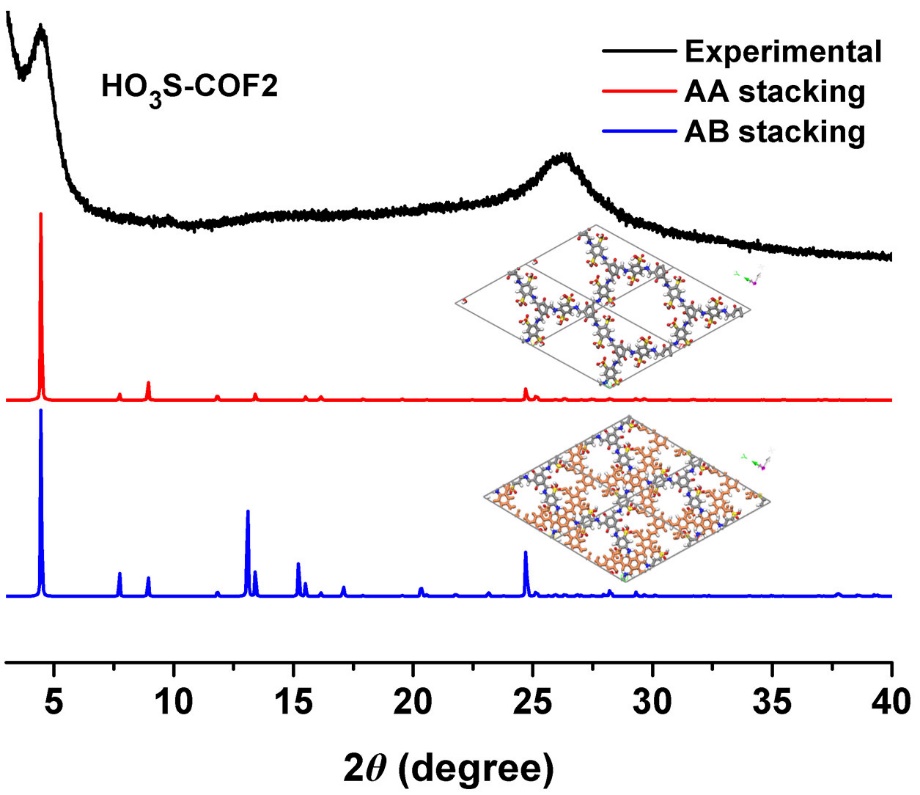


**Figure S7**. Calculated PXRD patterns based on the AA stacking, AB stacking and experimental result for HO_3_S-COF2.


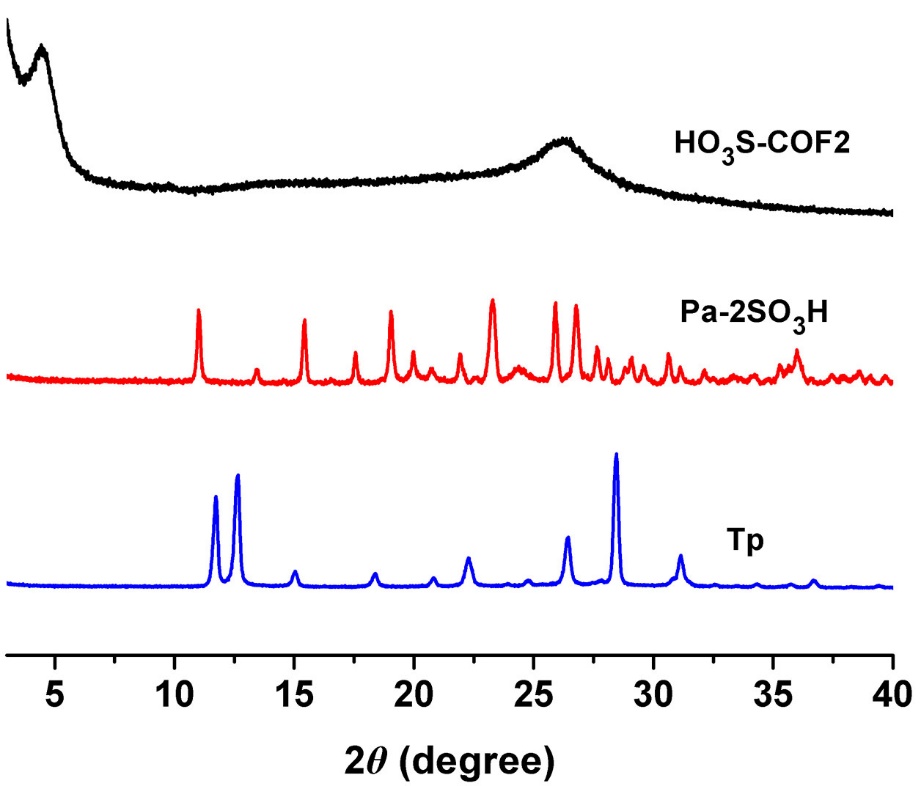


**Figure S8**. PXRD patterns of HO_3_S-COF2 and starting materials of Tp and Pa-2SO_3_H.


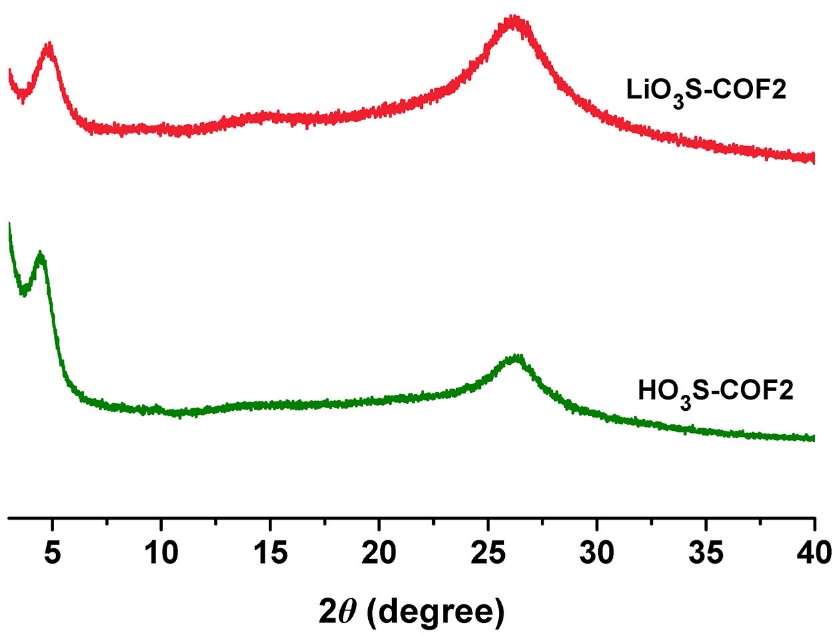


**Figure S9**. PXRD patterns of HO_3_S-COF2 and LiO_3_S-COF2.


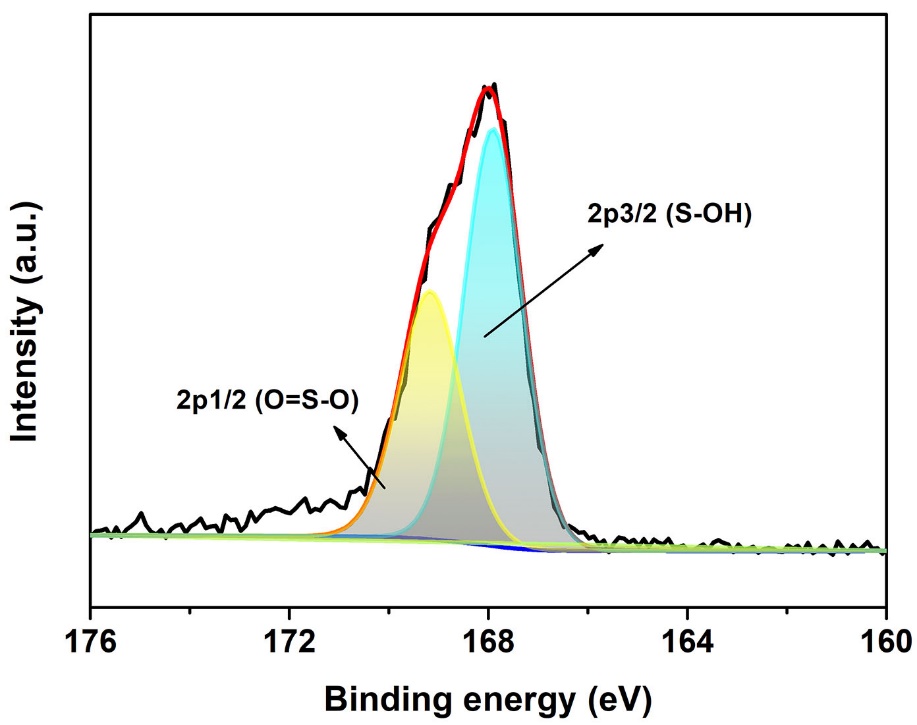


**Figure S10**. High-resolution XPS spectra of S 2p of HO_3_S-COF1.


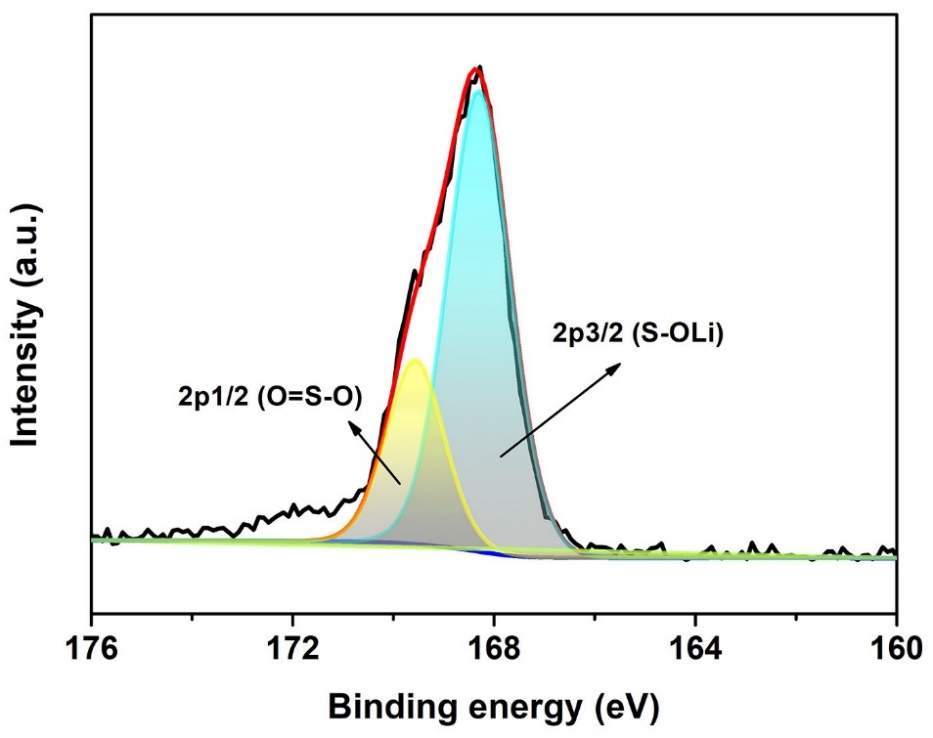


**Figure S11**. High-resolution XPS spectra of S 2p of LiO_3_S-COF1.


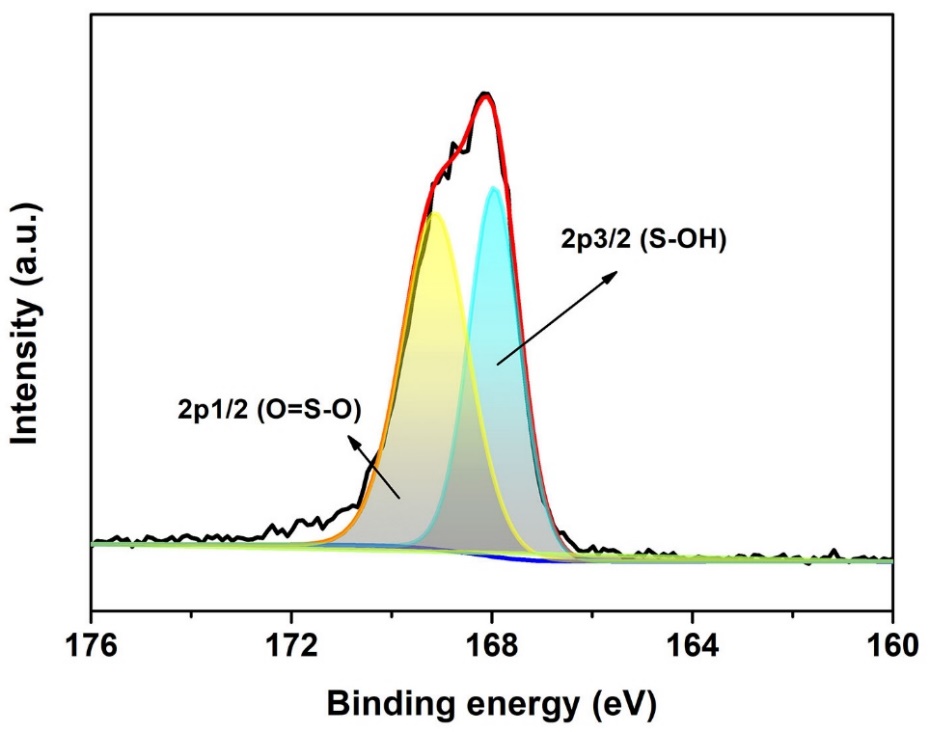


**Figure S12**. High-resolution XPS spectra of S 2p of HO_3_S-COF2.


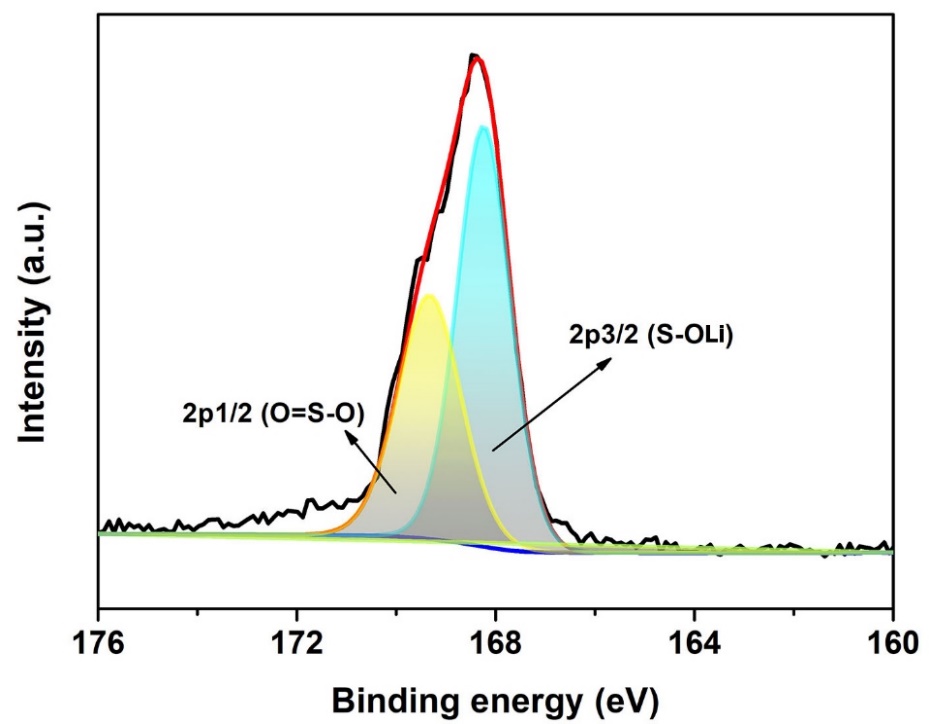


**Figure S13**. High-resolution XPS spectra of S 2p of LiO_3_S-COF2.


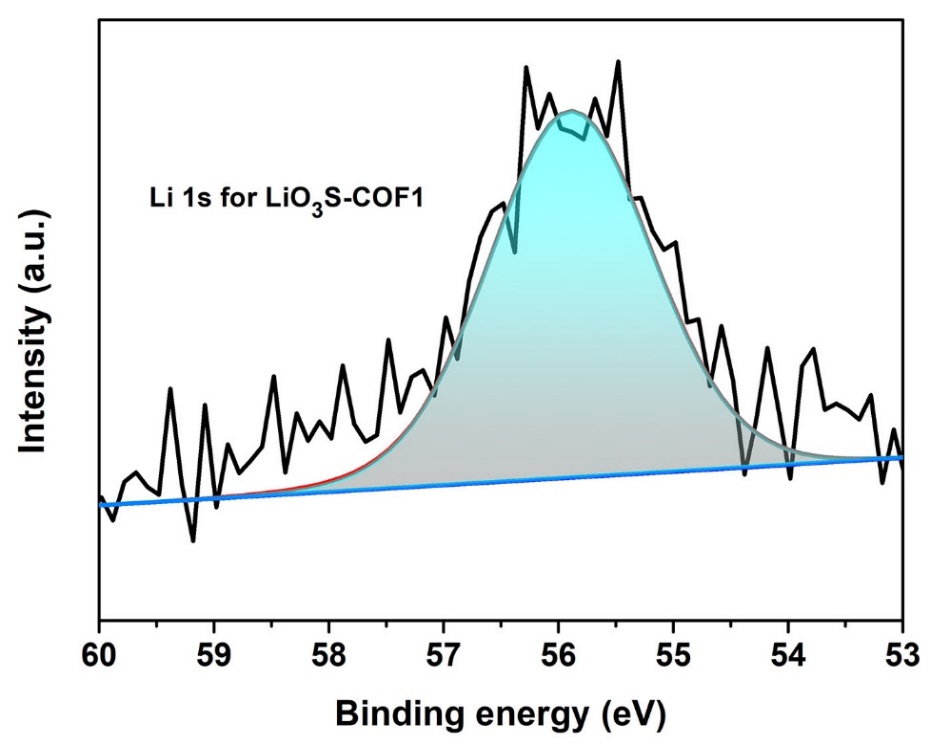


**Figure S14**. High-resolution XPS spectra of Li 1s of LiO_3_S-COF1.


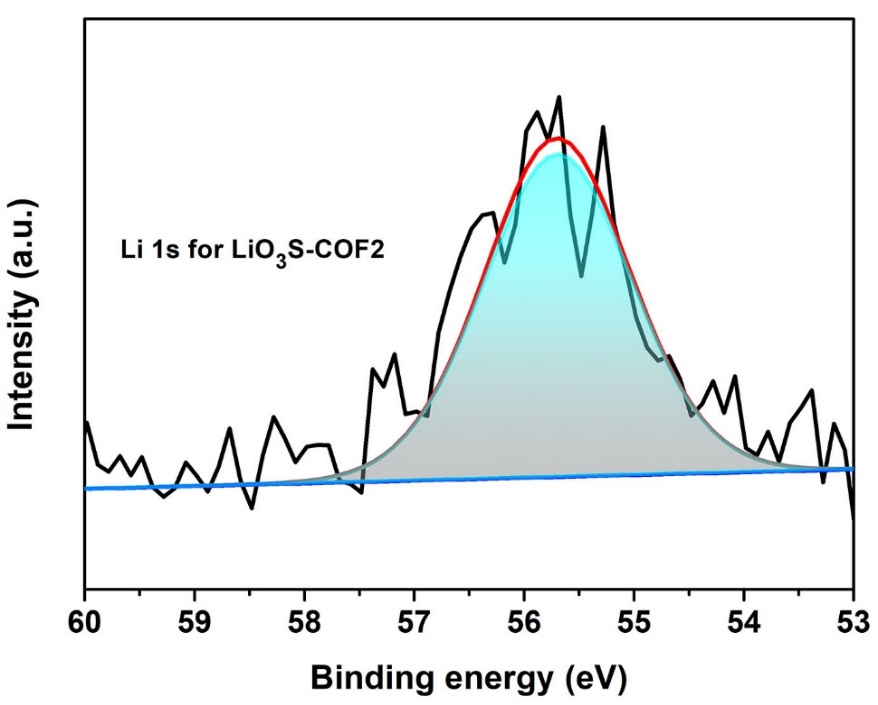


**Figure S15**. High-resolution XPS spectra of Li 1s of LiO_3_S-COF2.


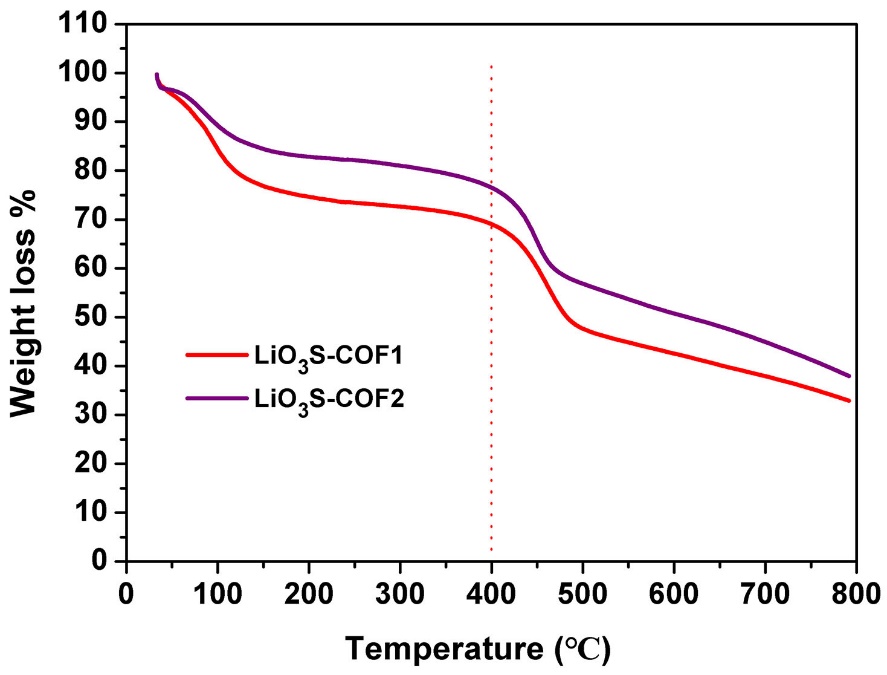


**Figure S16**. TGA curves of LiO_3_S-COF1 and LiO_3_S-COF2.


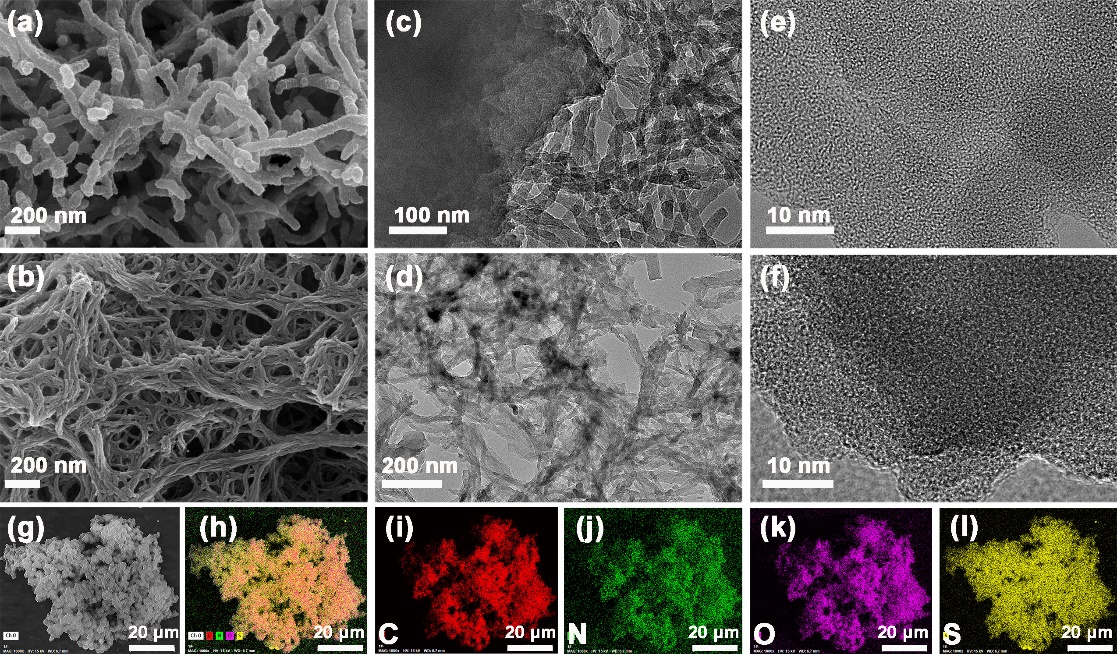


**Figure S17**. SEM images of HO_3_S-COF1 (a) and LiO_3_S-COF3 (b); TEM images of HO_3_S-COF1 (c) and LiO_3_S-COF1 (d); HRTEM images of HO_3_S-COF1 (e) and LiO_3_S-COF1 (f); EDS mappings of LiO_3_S-COF1 (g-l).


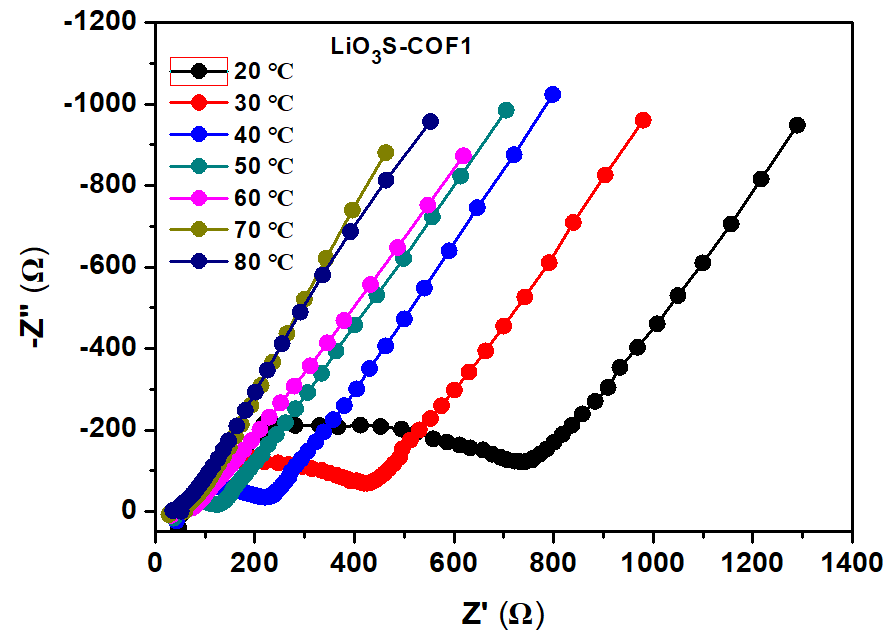


**Figure S18**. EIS of LiO_3_S-COF1 at various temperatures.


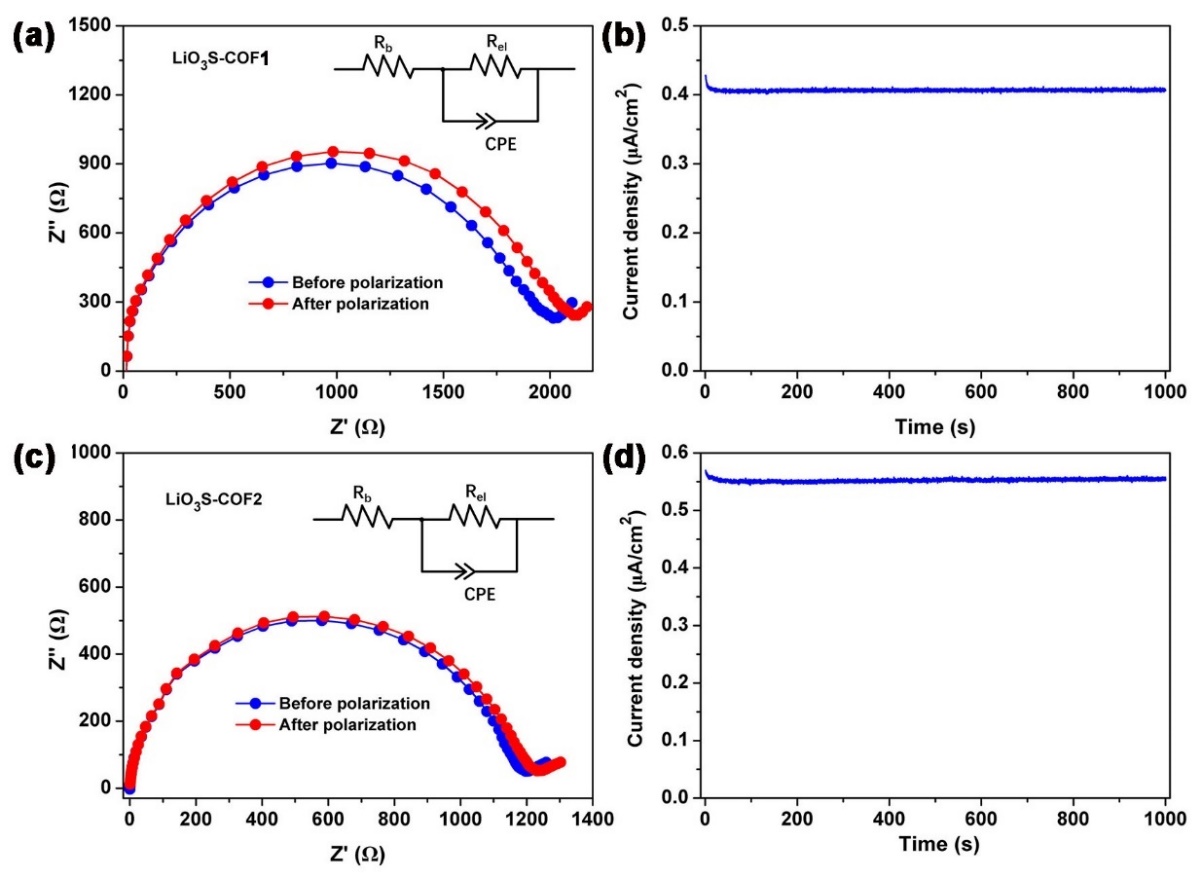


Figure S19. ESI (a-c) before and after polarization at 50 mV and the corresponding polarization curves(b-d) for LiO_3_S-COF1 and LiO_3_S-COF2. (Inset: simulating equivalent circuit, Rb, Rel stands for the electrolyte resistance and electrolyte/Li electrode interface resistance respectively, CPE is the constant phase element).


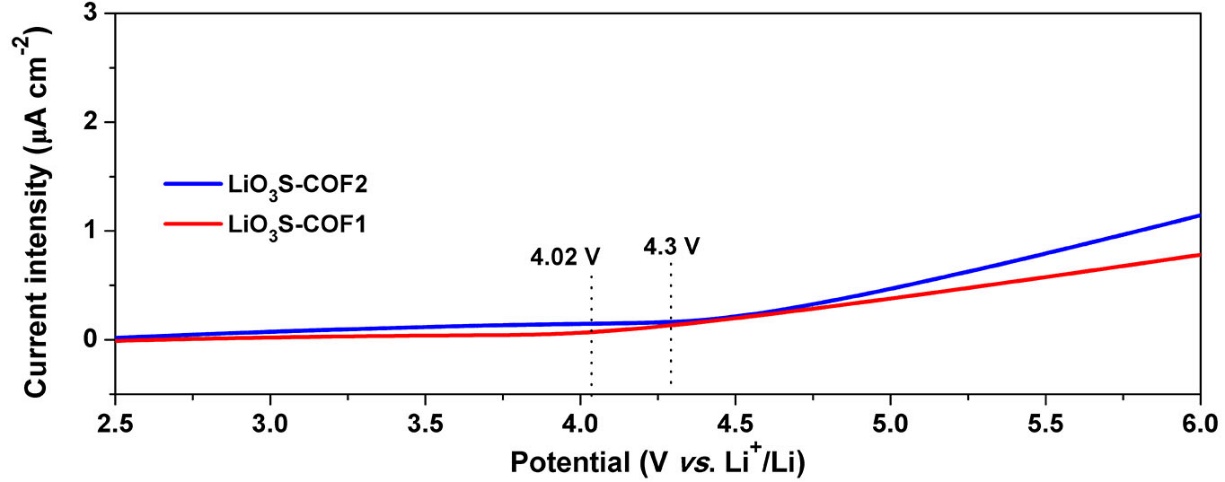


**Figure S20**. LSV study of LiO_3_S-COF2. The LiO_3_S-COF2 exhibits an electrochemical window as wide as 4.3 V than LiO_3_S-COF1 with 4 V according to published work,^[20]^ rivalling those of oxide ceramic based composite electrolyte.





**Figure S21**. FT-IR patterns of LiO_3_S-COF2 before and after tests in symmetric battery.





**Figure S22**. PXRD patterns of LiO_3_S-COF2 before and after tests in symmetric battery.





**Figure S23**. Galvanostatic charging-discharging curves of AQ | LiTFSI | Li and AQ | LiO_3_S-COF2 | Li cells at a current density of 500 mA g^−1^.





**Figure S24**. Charging-discharging profiles of AQ | LiO_3_S-COF2 | Li at different current densities.





**Figure S25**. Rate performances of AQ | LiTFSI | Li and AQ | LiO_3_S-COF2 | Li cells at various current densities.


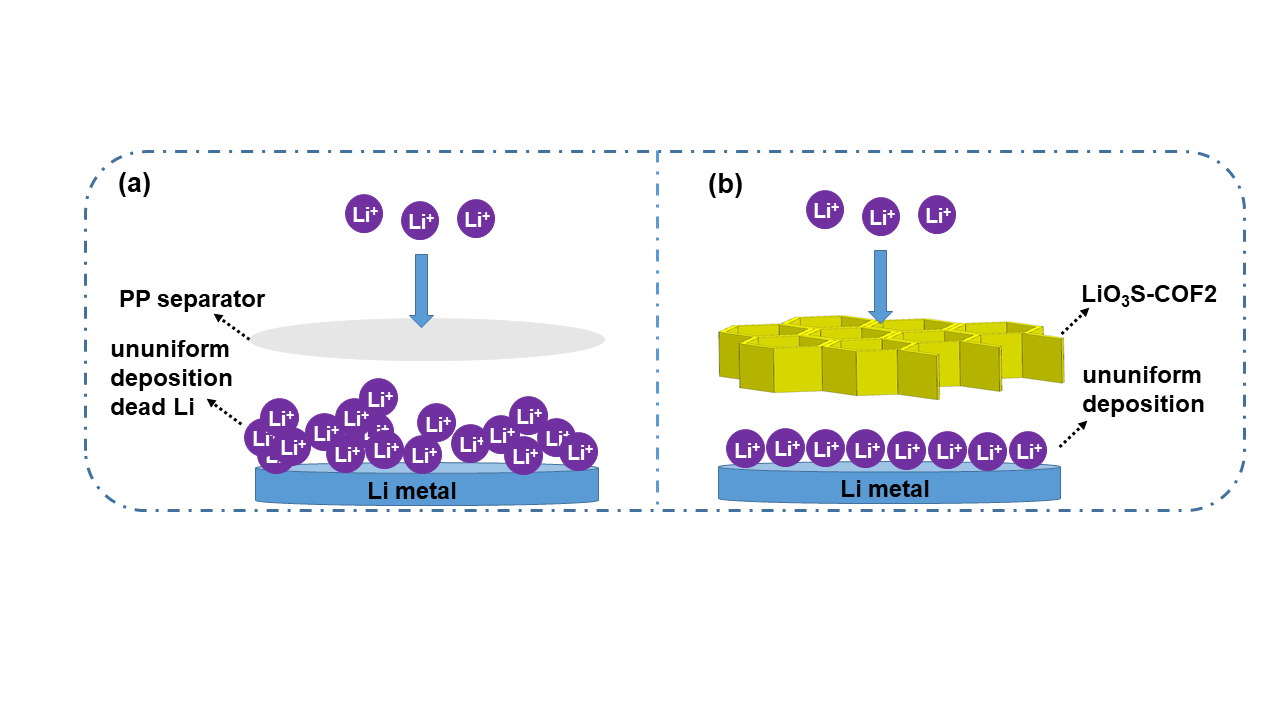


**Figure S26**. Depiction of the Li^+^ ununiform deposition process on the surface of Li metal by PP separator (a) and uniform deposition on functionalized LiO_3_S-COF2 (b).


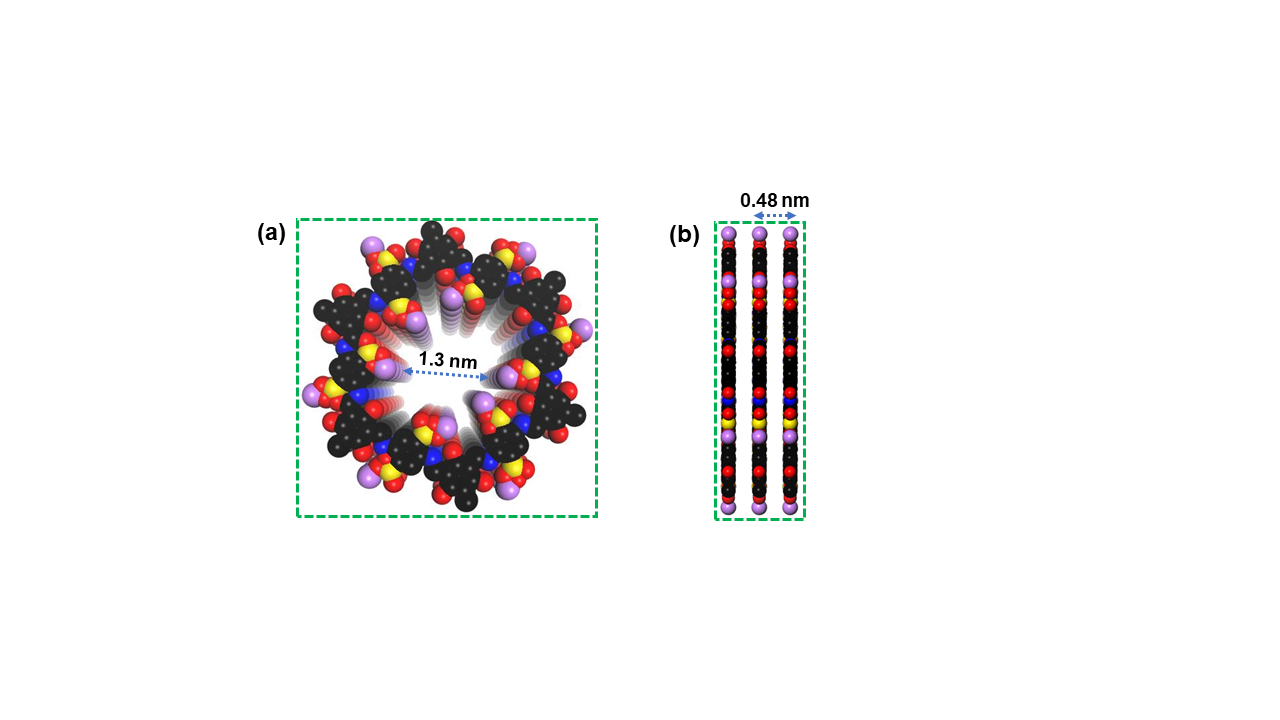


**Figure S27**. Optimized Li-ion geometries in LiO_3_S-COF2, which shows the values of pore diameter and interplanar distance, respectively.


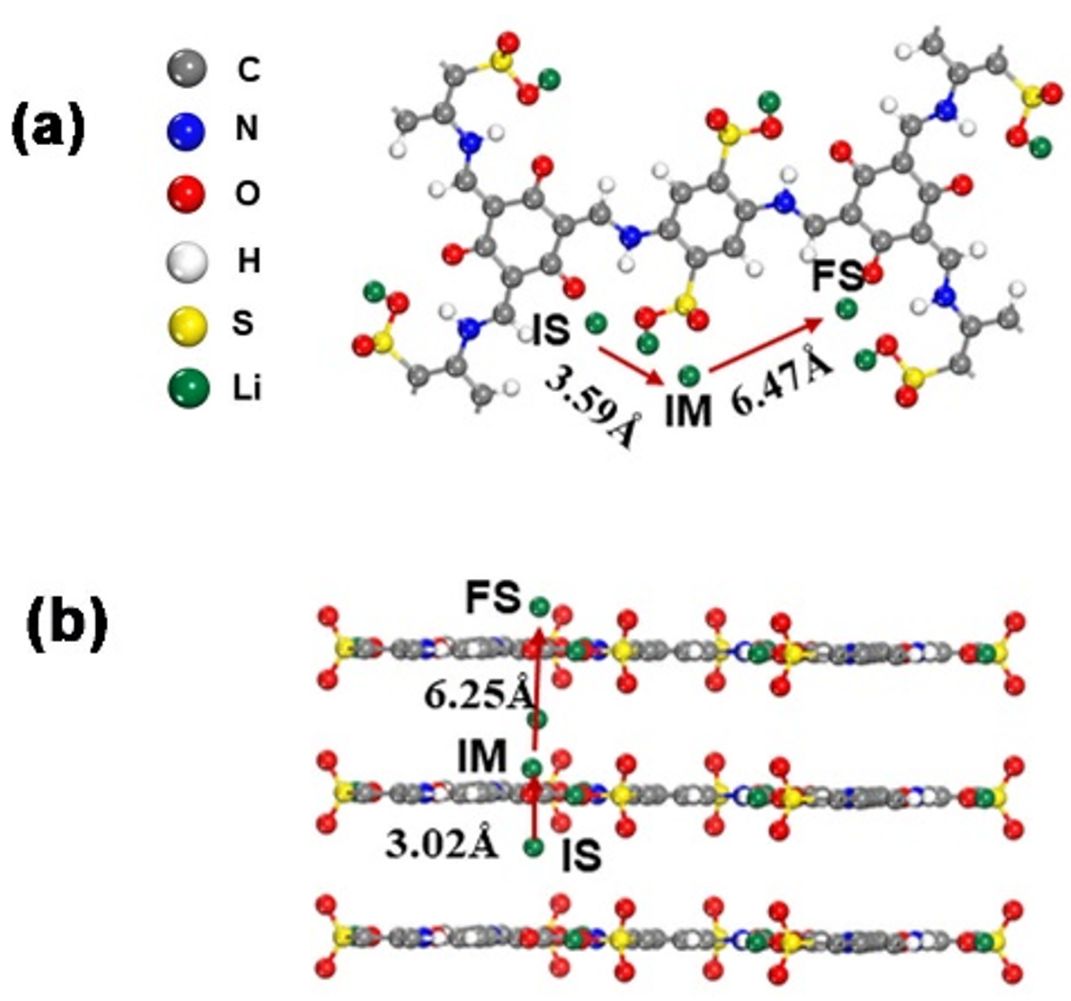


**Figure S28**. Li^+^ hopping distances (Å) in the (a) planar and (b) axial pathways in LiO_3_S-COF2.

**Table S1** Electrochemical performance comparison of LiO_3_S-COF1 and LiO_3_S-COF2.

| COFs | | LiO_3_S-COF1 | LiO_3_S-COF2 |
| --- | --- | --- | --- |
| impedance (Ω) | 20 | 750 | 450 |
|  | 30 | 430 | 365 |
|  | 40 | 225 | 202 |
|  | 50 | 130 | 122 |
|  | 60 | 85 | 81 |
|  | 70 | 67 | 62 |
|  | 80 | 59 | 40 |
| σ (S cm^−1^) | 20 | 2.67×10^−5^ | 4.46×10^−5^ |
|  | 30 | 4.65×10^−5^ | 5.47×10^−5^ |
|  | 40 | 8.89×10^−5^ | 9.9×10^−5^ |
|  | 50 | 1.53×10^−4^ | 1.63×10^−4^ |
|  | 60 | 2.36×10^−4^ | 2.47×10^−4^ |
|  | 70 | 2.98×10^−4^ | 3.23×10^−4^ |
|  | 80 | 3.39×10^−4^ | 5.06×10^−4^ |
| Ea (eV) | | 0.17 | 0.15 |
| *t*_Li⁺_ | | 0.91 | 0.93 |

**Table S2.** Electrochemical performances comparison of our work with reported works.

| COFs | EW (V) | σ (S cm^−1^)  (30 °C) | Ea (eV) | | | AC | | *t*_Li_ | Ref |
| --- | --- | --- | --- | --- | --- | --- | --- | --- | --- |
| LiCON-COF | 3.8 | 5.7×10^-5^  (25 ℃) | 0.34 | LiTFSI | | | 0.61 | | 9 |
| Hydrazone-linked COF nanosheets | 4.5 | 3.21 × 10^–5^ (20 ℃) | 0.13 | **−** | | | 0.92 | | 10 |
| Im-COF-TFSI | 4.2 | 2.9 × 10^–5^ | 0.32 | | LiTFSI | | 0.62 | | 11 |
| dCOF-ImTFSI | ~5.3 | 7.05 × 10^−3^ (150°C) | 0.054 | ImTFSI | | | 0.72 | | 12 |
| H-Li-ImCOF | 4.0 | 7.2 × 10^–3^ | 0.1 | PC | | | 0.91 | | 13 |
| Hydrazone-linked COF-PEO | 4.2 | ~1.0 × 10^–6^  (20 °C) | N/A | LiTFSI | | | N/A | | 14 |
| Triboronate ester-linked COF | N/A | 2.6 × 10^–4^  (25 °C) | 0.037 | | | LiClO_4_ | N/A | | 15 |
| Imine-linked COF-PEO-3 | N/A | 6.04 × 10^–6^  (40 °C) | 0.87 | LiClO_4_ | | | N/A | | 16 |
| Ge-COF-1 | N/A | 6.04 × 10^–6^ | 0.29 | EC/DEC | | | 0.67 | | 17 |
| Tp-PaSO_3_Li-COF | N/A | 1.6 × 10^–3^  (20 °C) | 0.13 | EC/DEC | | | 0.94 | | 18 |
| Cyclodextrin based COF | N/A | 2.7 × 10^–3^ | 0.26 | LiPF_6_,  EC/DMC | | | N/A | | 19 |
| Tp-PaSO_3_Li | 4.0 | 2.7 × 10^–5^  (25 °C) | 0.18 | **−** | | | 0.9 | | 20 |
| Cationic COF/PE | 4.2 | 1.62 × 10^–4^ | N/A | LiTFSI | | | 0.32 | | 21 |
| Ionic spiroborate-linked COF | <4.0 | 3.05 × 10^–5^  (25 ℃) | 0.24 | PC | | | 0.8 | | 22 |
| Q-COF | 5.6 | 7.5×10^-5^ | 0.19 | LiTFSI | | | 0.72 | | 3 |
| **LiO_3_S-COF2** | **4.3** | **5.47×10^-5^** | **0.15** | **−** | | | **0.93** | | **work** |

Abbreviations: EW = electrochemical window; AC = additional components; EC = ethylene carbonate; DMC = dimethyl carbonate; PC = propylene carbonate; NMP = N-methyl-2-pyrrolidinone; TFSI^–^ = bis(trifluromethanesulfonyl)imide.

**Table S3**. Unit cell parameters for LiO_3_S-COF2 and fractional atomic coordinates for the unit cell of LiO_3_S-COF2.

| Space group | | *P1* | |
| --- | --- | --- | --- |
| Symmetry | | triclinic | |
| Calculated unit cell | | *a*=18.4229 Å, *b*=19.4139 Å, *c*=4.8290 Å  *α*=*β*=90°*, γ*=111.3029° | |
| Atom list | x | y | z |
| H1 | 4.028104 | 1.168431 | -0.6004 |
| H2 | 3.705888 | 1.026662 | -0.27233 |
| H3 | 3.900632 | 0.840979 | -0.34658 |
| H4 | 3.824793 | 1.177115 | -0.46273 |
| H5 | 3.742136 | 0.800303 | -0.58402 |
| H6 | 3.772632 | 1.318647 | 0.274639 |
| H7 | 3.624624 | 1.087513 | -0.21967 |
| H8 | 3.638401 | 0.781759 | -0.21605 |
| H9 | 3.748547 | 0.612432 | 0.233333 |
| H10 | 3.579879 | 1.219616 | 0.559234 |
| H11 | 3.693797 | 1.374454 | 0.402247 |
| H12 | 3.491518 | 1.547409 | 0.27694 |
| H13 | 3.639669 | 1.596021 | 0.608941 |
| H14 | 3.354959 | 1.224613 | 0.569369 |
| H15 | 3.290403 | 1.339544 | 0.443632 |
| H16 | 3.093589 | 1.236017 | -0.24085 |
| H17 | 3.301447 | 1.16358 | 0.167557 |
| H18 | 3.103804 | 1.059494 | -0.51661 |
| Li1 | 3.980523 | 0.688651 | -0.52955 |
| Li2 | 3.368838 | 1.073346 | 0.404207 |
| Li3 | 3.122037 | 1.471421 | 0.087818 |
| Li4 | 3.292643 | 0.941527 | -0.20114 |
| Li5 | 3.936972 | 1.180893 | -0.22423 |
| Li6 | 3.362705 | 0.650401 | 0.254596 |
| C1 | 3.883555 | 1.076146 | -0.59816 |
| C2 | 3.807357 | 1.032295 | -0.47723 |
| C3 | 3.791829 | 0.952889 | -0.43117 |
| C4 | 3.858684 | 0.927204 | -0.4053 |
| C5 | 3.938437 | 0.984867 | -0.39543 |
| C6 | 3.952684 | 1.059934 | -0.51231 |
| C7 | 4.023558 | 1.114371 | -0.52571 |
| C8 | 3.757886 | 1.062844 | -0.37536 |
| C9 | 3.849635 | 0.855193 | -0.3787 |
| 10 | 3.737074 | 0.74759 | -0.19528 |
| C11 | 3.737881 | 1.17279 | -0.16317 |
| C12 | 3.778347 | 1.240969 | -0.03105 |
| C13 | 3.739847 | 1.268447 | 0.165765 |
| C14 | 3.661093 | 1.230327 | 0.231676 |
| C15 | 3.619934 | 1.163972 | 0.091109 |
| C16 | 3.658238 | 1.136284 | -0.10478 |
| C17 | 3.662987 | 0.745308 | -0.11658 |
| C18 | 3.619929 | 0.696656 | 0.088622 |
| C19 | 3.651098 | 0.648227 | 0.215855 |
| C20 | 3.725452 | 0.650991 | 0.139964 |
| C21 | 3.768346 | 0.699281 | -0.0664 |
| C22 | 3.638427 | 1.335445 | 0.472859 |
| C23 | 3.582659 | 1.362151 | 0.541145 |
| C24 | 3.597556 | 1.441622 | 0.498989 |
| C25 | 3.53213 | 1.464578 | 0.409956 |
| C26 | 3.455946 | 1.40474 | 0.352393 |
| C27 | 3.436744 | 1.330904 | 0.485704 |
| C28 | 3.501973 | 1.314297 | 0.610694 |
| C29 | 3.540155 | 1.535602 | 0.362377 |
| C30 | 3.364373 | 1.278843 | 0.484978 |
| C31 | 3.247549 | 1.245772 | 0.165911 |
| C32 | 3.188745 | 1.266 | 0.041807 |
| C33 | 3.138622 | 1.219356 | -0.15366 |
| C34 | 3.14649 | 1.152758 | -0.23686 |
| C35 | 3.206769 | 1.133687 | -0.11821 |
| C36 | 3.256115 | 1.179872 | 0.080306 |
| N1 | 3.77642 | 1.141479 | -0.35817 |
| N2 | 3.776236 | 0.795994 | -0.41714 |
| N3 | 3.623536 | 1.257863 | 0.442495 |
| N4 | 3.609811 | 1.597086 | 0.426293 |
| N5 | 3.298685 | 1.292186 | 0.372974 |
| N6 | 3.093374 | 1.104737 | -0.43585 |
| O1 | 3.930271 | 0.752295 | -0.02923 |
| O2 | 3.885111 | 0.701072 | -0.51791 |
| O3 | 3.505413 | 1.042874 | 0.326124 |
| O4 | 3.456565 | 1.153105 | 0.297232 |
| O5 | 3.106617 | 1.377458 | -0.04766 |
| O6 | 3.252697 | 1.420247 | 0.097874 |
| O7 | 3.888762 | 1.122407 | -0.77499 |
| O8 | 3.991102 | 0.970485 | -0.28778 |
| O9 | 3.724824 | 0.910206 | -0.40135 |
| O10 | 3.663998 | 1.486387 | 0.519363 |
| O11 | 3.489562 | 1.264882 | 0.780611 |
| O12 | 3.411212 | 1.415517 | 0.186975 |
| O13 | 3.15701 | 1.356065 | 0.432312 |
| O14 | 3.475412 | 1.076207 | -0.11933 |
| O15 | 3.869909 | 0.621129 | -0.07498 |
| O16 | 3.225339 | 1.036744 | -0.51993 |
| O17 | 3.152409 | 0.980013 | -0.1185 |
| O18 | 3.303103 | 1.034865 | -0.0721 |
| O19 | 3.892761 | 1.313949 | -0.4186 |
| O20 | 3.904421 | 1.375033 | 0.016835 |
| O21 | 3.952667 | 1.264781 | -0.01166 |
| O22 | 3.525248 | 0.779534 | 0.157982 |
| O23 | 3.512507 | 0.68827 | 0.506841 |
| O24 | 3.442685 | 0.642564 | 0.037122 |
| S1 | 3.517013 | 1.112712 | 0.150344 |
| S2 | 3.860129 | 0.693154 | -0.17335 |
| S3 | 3.175914 | 1.352312 | 0.122429 |
| S4 | 3.222695 | 1.049336 | -0.20467 |
| S5 | 3.879658 | 1.296246 | -0.10656 |
| S6 | 3.527225 | 0.700997 | 0.19283 |

**References**

1 S. Chandra, T. Kundu, K. Dey, M. Addicoat, T. Heine, R. Banerjee, *Chem. Mater*. **2016**, *28*, 1489.

2 J. Evans, C. A. Vincent, P. G. Bruce, *Polymer* **1987**, *28*, 2324.

3 C. Q. Niu, W. J. Luo, C. M. Dai, C. B. Yu, Y. X. Xu, *Angew. Chem. Int. Ed*. **2021**, *60*, 2–10.

4 W. Kohn, L. J. Sham, *Phys. Rev*. **1965**, *140*, A1133.

5 P. E. Blöchl, *Phys. Rev. B* **1994**, *50*, 17953.

6 G. Kresse, J. Furthmüller, *Phys. Rev. B* **1996**, *54*, 11169.

7 J. P. Perdew, K. Burke, M. Ernzerhof, *Phys. Rev. Lett*. **1996**, *77*, 3865.

8 G. Henkelman, B. P. Uberuaga, H. Jonsson, *J. Chem. Phys*. **2000**, *113*, 9901.

9 H. W. Chen, H. Y. Tu, C. J. Hu, Y. Liu, D. R. Dong, Y. F. Sun, Y. F. Dai, S. L. Wang, H. Qian, Z. Y. Lin, L. W. Chen, *J. Am. Chem. Soc*. **2018**, *140*, 896−899.

10 X. Li, Q. Hou, W. Huang, H. S. Xu, X. W. Wang, W. Yu, R. L. Li, K. Zhang, L. Wang, Z. X. Chen, K. Y. Xie, K. P. Loh, *ACS Energy Lett*. **2020**, *5*, 3498-3506.

11 Z. Li, Z.-W. Liu, Z.-J. Mu, C. Cao, Z. Li, T.-X. Wang, Y. Li, X. Ding, B.-H. Han, W. Feng, *Mater. Chem. Front*. **2020**, *4*, 1164-1173.

12 Z. Li, Z.-W. Liu, Z. Li, T.-X. Wang, F. Zhao, X. Ding, W. Feng, B.-H. Han, *Adv. Funct. Mater*. **2020**, *30*, 1909267.

13 Y. Hu, S. Huang, I. Sellinger, M. Ortiz, W. Zhang, N. Dunlap, S.-H. Lee, S. Wan, Y. Jin, S. Lu, *J. Am. Chem. Soc*. **2019**, *141*, 7518-7525.

14 G. Zhang, Y.-l. Hong, Y. Nishiyama, S. Bai, S. Kitagawa, S. Horike, *J. Am. Chem. Soc*. **2019**, *141*, 1227-1234.

15 D. A. Vazquez-Molina, G. S. Mohammad-Pour, C. Lee, M. W. Logan, X. Duan, J. K. Harper, F. J. Uribe-Romo, *J. Am. Chem. Soc*. **2016**, *138*, 9767-9770.

16 Q. Xu, S. Tao, Q. Jiang, D. Jiang, *J. Am. Chem. Soc*. **2018**, *140*, 7429-7432.

17 S. Ashraf, Y. Zuo, S. Li, C. Liu, H. Wang, X. Feng, P. Li, B. Wang, *Chem. - Eur. J*. **2019**, *25*, 13479-13483.

18 J. Li, F.-Q. Zhang, F. Li, Z. Wu, C. Ma, Q. Xu, P. Wang, X.-M. Zhang, *Chem. Commun*. **2020**, *56*, 2747-2750.

19 Y. Zhang, J. Duan, D. Ma, P. Li, S. Li, H. Li, J. Zhou, X. Ma, X. Feng, B. Wang, *Angew. Chem. Int. Ed*. **2017**, *56*, 16313-16317.

20 Y. Jung, S. H. Kim, Y.-H. Lee, S. K. Kwak, S.-Y. Lee, *J. Am. Chem. Soc*. **2019**, *141*, 5880-5885.

21 W. Sun, J. Zhang, M. Xie, D. Lu, Z. Zhao, Y. Li, Z. Cheng, S. Zhang, H. Chen, *Nano Lett*. **2020**, *20*, 8120-8126.

22 Y. Du, H. Yang, J. M. Whiteley, S. Wan, Y. Jin, S.-H. Lee, W. Zhang, *Angew. Chem. Int. Ed*. **2016**, *55*, 1737-1741.
