# Supplementary figures and images for: Understanding a Single-Li-Ion COF Conductor for Being Dendrite Free in a Li-Organic Battery
Source: Research (Wash D C). 2022 Oct 2;2022:9798582. doi: 10.34133/2022/9798582 (PMC9575471; doi:10.34133/2022/9798582)

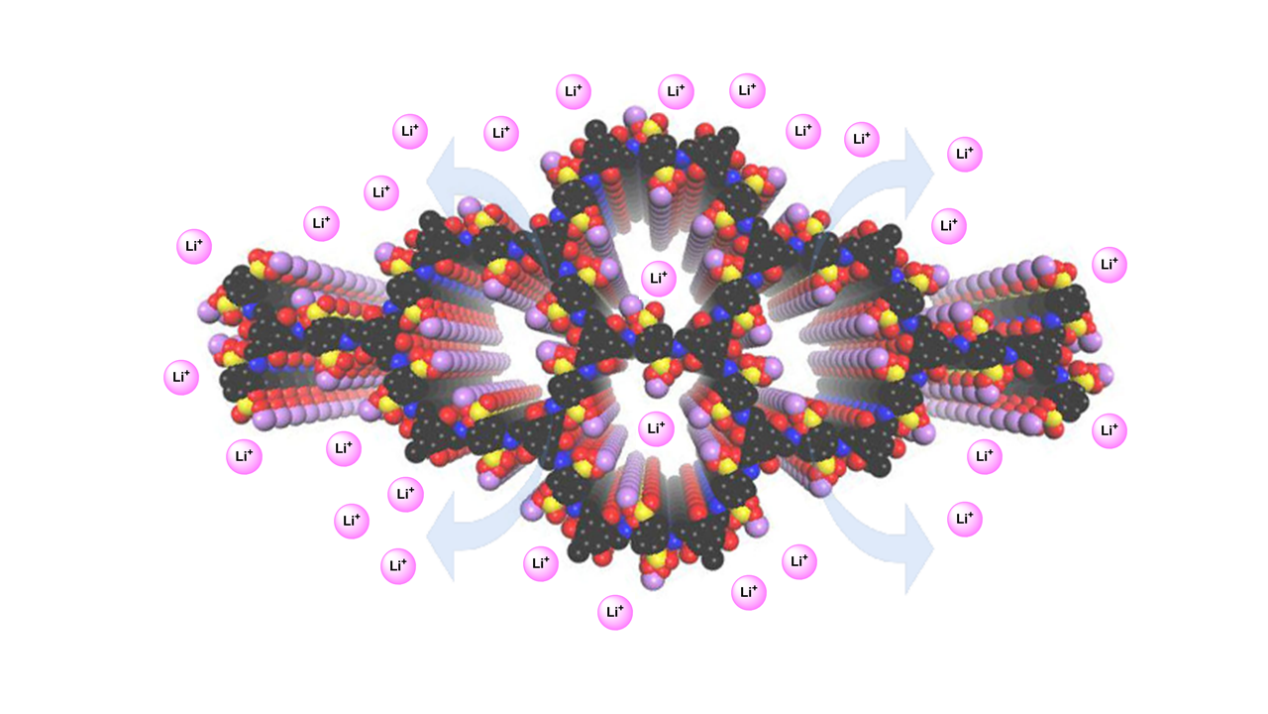


Schematic illustration of single Li-ion conductor based on covalent organic framework.

Supplement: Supplementary Materials — The chemical reagents, characterized instruments, electrochemical tests, DFT calculation details, tables, and other materials are provided in the supporting information. [file 9798582.f1.zip › TOC.docx]
